# Hazardous Materials from Threats to Safety: Molecularly Imprinted Polymers as Versatile Safeguarding Platforms

**DOI:** 10.3390/polym16192699

**Published:** 2024-09-24

**Authors:** Ana-Mihaela Gavrila, Aurel Diacon, Tanta-Verona Iordache, Traian Rotariu, Mariana Ionita, Gabriela Toader

**Affiliations:** 1 National Institute for Research, Development in Chemistry and Petrochemistry ICECHIM, 202 Spl. Independentei, 060021 Bucharest, Romania; ana.gavrila@icechim.ro (A.-M.G.); iordachev.icechim@gmail.com (T.-V.I.); 2 Military Technical Academy “Ferdinand I”, 39–49 George Cosbuc Boulevard, 050141 Bucharest, Romania; aurel_diacon@yahoo.com (A.D.); traian.rotariu@mta.ro (T.R.); 3 Advanced Polymer Materials Group, National University of Science and Technology POLITEHNICA Bucharest (UNSTPB), Gheorghe Polizu 1-7, 011061 Bucharest, Romania; mariana.ionita@polimi.it

**Keywords:** hazardous compounds, molecularly imprinted polymers, imprinting techniques, multi-stimuli responsive, sensing, decontamination, illicit drugs, explosives, CWA agents

## Abstract

Hazards associated with highly dangerous pollutants/contaminants in water, air, and land resources, as well as food, are serious threats to public health and the environment. Thus, it is imperative to detect or decontaminate, as risk-control strategies, the possible harmful substances sensitively and efficiently. In this context, due to their capacity to be specifically designed for various types of hazardous compounds, the synthesis and use of molecularly imprinted polymers (MIPs) have become widespread. By molecular imprinting, affinity sites with complementary shape, size, and functionality can be created for any template molecule. MIPs' unique functions in response to external factors have attracted researchers to develop a broad range of MIP-based sensors with increased sensitivity, specificity, and selectivity of the recognition element toward target hazardous compounds. Therefore, this paper comprehensively reviews the very recent progress of MIPs and smart polymer applications for sensing or decontamination of hazardous compounds (e.g., drugs, explosives, and biological or chemical agents) in various fields from 2020 to 2024, providing researchers with a rapid tool for investigating the latest research status.

## 1. Introduction

This review wraps together the recent literature findings in molecularly imprinted polymers (MIPs) as innovative smart polymeric platforms designed for the detection and decontamination of hazardous substances, including chemical warfare agents, explosives, illicit drugs, and biological agents. The overview comprises recent studies targeting the previously mentioned threat agents, along with their detection and decontamination methods.

### 1.1. Types of Hazardous Compounds

In broad terms, hazardous materials (HAZMAT) refer to substances that are flammable, explosive, poisonous, or radioactive [1]. Hazardous materials have the potential to cause harm to living organisms by damaging their tissues or disrupting crucial biological processes. The hazardous components most commonly found in the environment can be categorized into two groups: naturally occurring pollutants (can be found in the air, minerals, water, and soil) and anthropogenic pollutants (typically originate from combustion, chemical reactions, or the unsecured discharge of toxic materials) [2]. In the book *Hazardous Materials: Managing the Incident*, Noll G. et al. [3] have provided a series of definitions related to hazardous materials (HAZMAT):

- **Hazardous materials,** in a broad sense, refer to any substance or material, regardless of its form or quantity, that presents an unreasonable risk to safety, health, and property [3]. **Hazardous materials** are defined either as matter (solid, liquid, or gas) or energy form, which, when released, have the potential to cause harm to individuals, the surrounding environment, and property, including weapons of mass destruction (WMD), as well as any other criminal use of hazardous materials, such as illicit laboratories, environmental crimes, or industrial sabotage [3];
- **Hazardous substances**—any substance posing a threat to waterways and the environment when released [3];
- **Common hazardous materials** include nitrogen oxides, sulfur oxides, carbon oxides, hydrogen sulfide, volatile organic compounds (VOCs), nitrogen-containing compounds (NCCs), sulfur-containing compounds (SCCs), dyes, pharmaceuticals, and personal care products (PPCPs), etc. [2];
- **Volatile organic compounds (VOCs)**—chemicals with high vapor pressure, often emitted from solvents, resins, paints, adhesives, and similar substances. Hazardous VOCs include benzene, naphthalene, toluene, phenolics, xylenes, and similar compounds, posing risks to both the environment and human health [2];
- **Hazardous chemicals**—any chemical that would be a risk to employees if exposed in the workplace [3];
- **Dangerous goods**—in international transportation, hazardous materials are commonly referred to as “dangerous goods” [3];
- **Hazardous drugs**—medication used to treat illnesses such as cancer, arthritis, multiple sclerosis, and viral diseases possessing one/more of the following properties: carcinogenicity, reproductive toxicity, teratogenicity, genotoxicity, organ toxicity at low doses [3];
- **Illicit drugs**—legally produced drugs that are abused and drugs produced for no reason other than abuse are called abused drugs, drugs of abuse, or illicit drugs. In addition to legally produced pharmaceutical drugs, there are also substances that have no legitimate, recognized medicinal purpose but are produced and ingested entirely for their psychoactive effects [4];
- **Extremely Hazardous Substances (EHS)**—extremely hazardous to a community during a spill or release due to their toxicities and physical/chemical properties [3];
- **Hazardous wastes**—discarded materials regulated by the authorities due to public health and safety concerns [3];
- **Weapons of Mass Destruction (WMD)**—(1) any destructive device, such as any explosive, incendiary, or poison gas bomb, grenade, rocket having a propellant charge of more than four ounces, missile having an explosive or incendiary charge of more than one-quarter ounce (7 g), mine, or device similar to the above; (2) any weapon involving toxic or poisonous chemicals; (3) any weapon involving a disease organism; or (4) any weapon that is designed to release radiation or radioactivity at a level dangerous to human life [3];

A weapon of mass destruction is typically referred to as a chemical, biological, radiological, nuclear, or other device that is intended to cause harm to a significant number of individuals. The existence of CBRNE threats (including chemical, biological, radiological, or nuclear agents and explosive materials) poses supplementary concern due to their potential to cause severe harm and widespread contamination. Since these pollutants pose serious dangers to public health, the environment, and national security, it is imperative that all countries take the necessary precautions and take appropriate action to mitigate their effects.

**Chemical warfare agents (CWAs)** are a group of hazardous compounds that have been defined in the Convention on the Prohibition of the Development, Production, Stockpiling and Use of Chemical Weapons and their Destruction (Chemical Weapons Convention or CWC [5]) as any chemical substance whose toxic properties can cause death, temporary incapacitation or permanent harm to humans or animals. Although not included in Schedule 1 of the Convention, it is important to recognize that toxic substances and explosive precursors, including Toxic Industrial Materials (TIM) like ammonia, chlorine, cyanogen chloride, and phosgene, can present significant risks. These substances have been extensively researched and documented, underscoring the importance of understanding their potential hazards.

**Biological warfare agents (BWAs)** entail the deliberate release or threat of release of biological agents (such as viruses, bacteria, fungi, or toxins) with the intent to induce disease or mortality among human populations or to impact food crops and livestock in a manner that induces fear among civilian populations or influences governmental structures, being capable of causing fatal or chronic illnesses, or may even lead to epidemics. Biological agents are categorized into three groups: **(A)** high-priority pathogens like *Bacillus anthracis*, *Clostridium botulinum* toxin, *Variola major*, and filoviruses such as *Ebola*; **(B)** consisting of second highest-priority bio-agents including *Brucella species*, *Salmonella* species, *Escherichia coli*, *Chlamydia psittaci*, and ricin; **(C)** encompassing emerging pathogens that could be engineered for mass dissemination, such as *Nipah* virus, *Hantavirus*, *Yellow fever* virus, and multi-drug-resistant tuberculosis [6].

**Ionizing radiation** presents a substantial risk to human health and life due to its impact on cell metabolism, disruption of organism functioning, and potential fatality. There are two types of ionizing radiation: electromagnetic radiation (gamma, X-ray, and UV radiation) and corpuscular radiation (alpha and beta particles, protons, neutrons, and fragments of heavy atomic nuclei). Radiological agents can have detrimental effects on DNA synthesis, leading to adverse impacts on blood, reproductive organs, and young cells [7].

**Nuclear weapons** are based on nuclear reactions having the potential to cause widespread devastation and loss of life. Disarmament is the best protection against these catastrophic weapons. Several treaties aim to prevent nuclear proliferation and testing [7]. 

### 1.2. Hazardous Compounds Assessment Strategies and Designed Polymeric Platforms

Multiple efforts have been made to address exceptionally high risks related to hazardous compounds, which need specially tailored management methods and risk-reduction strategies, particularly for environmental applications. Some specific actions that are essential when encountering hazardous materials threats include detection, identification, neutralization, removal, and disposal. Detection and identification measures commonly combine traditional methods, including liquid chromatography (LC) and gas chromatography (GC), coupled with either mass spectrometry (MS) or tandem mass spectrometry (MS/MS). Nonetheless, these techniques present some limitations, such as expensive materials or equipment, usually requiring pre-treatment liquid-liquid extraction or solid phase extraction (SPE) steps, and the results could be false by the presence of other interferents.

Any HAZMAT management system must implement effective procedures for reducing, detecting, and removing contaminants. Advanced polymeric innovative platforms have been engineered to detect and eliminate hazardous contaminants. The broad applications of smart materials in catalysis, sensing, and decontamination methodologies make them highly relevant in addressing HAZMAT and environmental challenges.

Expanding and enhancing current applications would be possible by developing novel polymers and cross-linking agents that have better biodegradability and biocompatibility. The most captivating aspect of smart polymers lies in their ability to be customized and their adaptable sensitivity [8]. 

**Figure 1:**
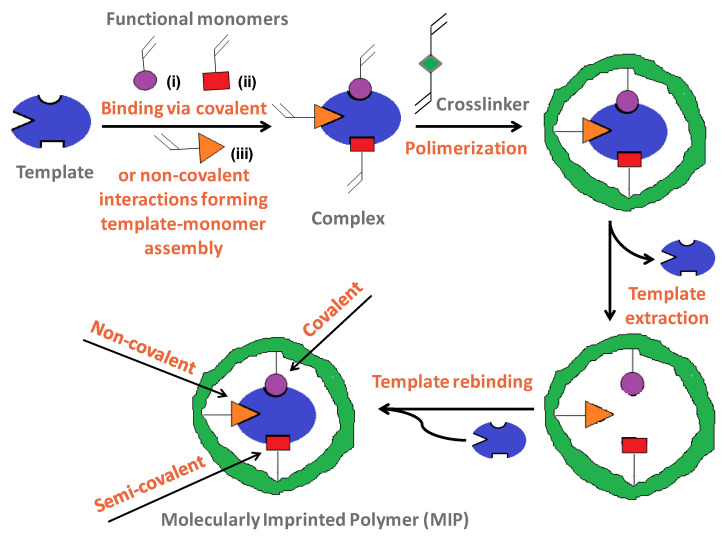
Schematic representation of the synthesis of an MIP by covalent, semi-covalent, and non-covalent bonds (adapted from [13]).

Among historically bioinspired advanced polymeric platforms, Molecularly Imprinted Polymers (MIPs) are an attractive alternative to traditional analyzing methods, encompassing main applications including sample pretreatment (solid-phase extraction SPE), chromatographic separation, sensing, catalysis, and monitoring/diagnostic. Since the first use of the term “molecular imprinting” (MI) in 1931 by M. V. Polyakov [9] and the pioneering studies on organic MIPs in 1972 by Wulff and Sarhan [10], MI technique [11] has proved to be a tailor-made fashion technique for designing synthetic and smart antibodies, which generate guest-binding activity and selectivity. In a typical approach, the MI process (Figure 1) allows the creation of specific complementary cavities of the same shape and size but with complementary electronic entourage by the polymerization of a functional monomer in the presence of target molecules (called template) and of a high concentration of crosslinker. These imprinted cavities allow only similar structures to be specifically retained in the polymer, from which they can be later on removed by heating or washing with solvent without altering the geometry of the polymer (conferring reusability to the employed material) [12]. Other advantages brought by the use of MIPs refer to higher selectivity, sensitivity, and low detection limits, but also to ease of preparation, storage stability, cost-effectiveness, high mechanical strength, and resistance to heat and pressure, as well as to harsh chemical environments. 

A stable complex is formed between the template and functional monomer(s) by covalent (Figure 1—monomer i), semi-covalent (Figure 1—monomer ii), and non-covalent bonds, the non-covalent approach (Figure 1—monomer iii) being preferred thanks to its simplicity and the ease of the template extraction or using readily available functional monomers. A reversible covalent bond is formed between functional monomers and template molecules, leading to a very uniform distribution of binding sites, while the semi-covalent bond carries both other bonds. In the non-covalent case, polymerization takes place in the presence of an initiator and a porogen solvent via hydrogen bonding, van der Waals forces, π-π interactions, and/or hydrophobic or ionic bonds interactions; the latter has the role of creating pores in the polymer matrix that facilitate the access of target molecules near the imprinted sites, during the rebinding assays.

In this regard, an overview related to modern and dedicated methods applied to produce MIPs, mostly for sensing applications, by surface polymerization, electropolymerization, sol-gel derived techniques, phase inversion, and deposition of electroactive pastes/inks incorporating MIP particles, was already provided by our group [13]. Many literature papers or reviews have dealt with general polymerization technologies; therefore, they will not be described here.

Alongside surface polymerization, nanoscale imprinting is a smart strategy based on the incorporation of fluorescent or electrochemical nanomaterials during the synthesis process of MIPs, such as quantum dots (QDs), carbon nanotubes (CNTs) and graphene-derived nanomaterials, rare-earth metal-based nanoparticles, metal (Au, Pt, Ni) nanoparticles/nanowires or PtAu, AuAg bimetallic, magnetic nanoparticles, and metal–organic frameworks (MOFs) [14].

These nanomaterials have gained considerable attention during the last years thanks to their excellent electronic, optical, and electrocatalytic attributes. Therefore, molecularly imprinted nanoparticles (nanoMIPs) have proven to be powerful tools for molecular therapy/cellular studies/drug delivery [15], particularly when integrated into various transducers via sensing applications [16]. Noteworthy is the fact that nanostructured MIPs are expected to improve the binding properties and site accessibility of imprinted materials, boosting both the sensitivity (due to nanomaterials) and the selectivity (due to MIPs), especially for biosensing applications. Metal–organic frameworks (MOFs) are crystalline three-dimensional materials composed of metal ions or clusters (usually transition metal ions, such as copper, zinc, aluminum, etc.) surrounded by organic ligands (usually organic molecules containing functional groups, such as carboxylic acid, pyridine) connected through coordination bonds [17]. The cage-like structures endow MOFs with unique characteristics, including high surface area, controllable and uniform pore structure, flexible geometry, and significant thermal stability. Despite their excellent properties, MOFs cannot accurately identify target molecules and lack specific recognition ability; thus, MOFs and MIPs have been lately used for recognizing hazards or contaminants in food, drug delivery, or environmental applications [18].

Among the main aforementioned applications of the MIPs, this review focuses on MIPs used as recognition elements for developing sensing/diagnostic devices for different hazardous compounds that are ultimately used for environmental safety applications or decontamination purposes. With the unique recognition mechanism, MIPs can recognize various extremely dangerous targets such as chemical warfare agents, explosives, illicit drugs, and biological or other harmful agents, which basically cover all categories of HAZMAT.

(Bio/chemo)sensors are defined as analytical nanotech devices that convert a biological response into a quantifiable and measurable signal (detecting small to large analytes present in various samples including body fluids, food, cell cultures, environmental or HAZMAT). *Electrochemical biosensors* have been assumed as vying crucial alternatives as they are stimulated by their quick reaction and are determined by potentially cheaper, simple to use, and portable characteristics over conventional ones, excellent sensitivity, and low limits of detection, as well as their precision and specificity. Moreover, they offer good prospects for meeting the growing needs in sensing applications of various biomarkers, such as proteins, nucleic acids, and lipids, as well as several types of hazardous compounds [19]. Despite these numerous advantages, the selectivity of sensors still needs improvement, and MIPs are quite promising in overcoming this shortcoming. MIP-based sensing (electrochemical, optical) is one of the most promising emerging technologies that can identify and quantify the target species by converting the hazards-MIP binding event into a physically or chemically readable signal. In the case of electrochemical MIP sensors [20], the signal is translated into a measurable output such as current (voltammetric or amperometric sensors), potential (potentiometric sensors), conductivity (conductometric sensors), and impedance (impedimetric sensors) change by the transducer, with in-situ/real-time detection via good limits of detection (LOD) and limits of quantification (LOQ). 

Various kinds of electrodes found application for electrochemical/optical detection of hazards-type compounds, including glassy carbon electrodes (GCEs), platinum, gold, or mercury-film deposited electrodes, graphene-type electrodes, fluorescent materials such as quantum dots (QDs) and screen-printed electrodes (SPEs) such as carbon, platinum or gold SPEs. SPEs are the most used and are lightweight instruments, leading to cost-effective sensors because of their well-known and established technology for automation (can be further turned into point-of-care POC devices), using small sample volumes (~2–100 μL) or high temperature for analyte detection [13,20]. As for the assembly process of MIPs, there are several common methods to modify the electrode surface with an MIP [13,20]: (i) by electrochemical polymerization directly in a solution containing templates and electroactive functional monomers; (ii) drop-casting of pre-prepared polymers from solution; (iii) spin coating of a complex containing functional monomers and templates chemically polymerized in situ in solution; (iv) grafting polymerizable groups and/or initiators onto the support surface; (v) layer-by-layer self-assembly on the transducer surface; (vi) three-dimensional (3D) printing for smart materials such as inkjet printing/tip-based printing based on single droplet manipulation and 3D printing/electrohydrodynamic printing based on continuous droplet manipulation [21].

Sensors with nanoMIP as a recognition element possess outstanding attributes, including molecular specificity, robustness, low cost and reusability, physiochemical/thermal stability in harsh chemical conditions, ease of preparation, and automation via computer-designed methods. Other MIP sensors for HAZMAT are considered mass-sensitive sensors (quartz crystal microbalance QCM or surface acoustic wave SAW sensors) that generate their signal thanks to a mass change, leading to a measurable, reliable, and analytical frequency response [22].

Besides electrochemical sensors, optical sensing techniques with MIP nanomaterial-based concepts for hazardous compounds covering absorption, fluorescence, surface plasmon resonance (SPR), surface-enhanced Raman scattering (SERS) signal, and color change have been explored [23]. For luminescent optical sensors, the detection method relies on the conversion of a binding event into a measurable light thanks to fluorescence, chemiluminescence, or colorimetric changes; these light signal changes lead to highly sensitive, flexible, and inexpensive sensors. The signal transduction of optical MIP sensors can be translated by the optical activity of the target, incorporation of a fluorophore or a chromophore into the polymer matrix, and upon a catalytic reaction, leading to spectroscopically active species. Because the MIPs-based optical sensors use fewer reagents and smaller volumes of sample, the specificity of these devices can be quite low, while the instability raises difficulties for real-time monitoring.

This review is focused on the very recent advances in MIPs as versatile and ideal platforms for both detection and decontamination of a variety of HAZMAT ranging from chemical warfare agents, explosives, illicit drugs, biological or other agents, from complex matrices or real toxic samples. We described the most recent trends in MIP-based optical and electrochemical sensors for HAZMAT detection and decontamination or treatments, discussing in detail their characteristics and limitations. 

### 1.3. Green Aspects of MIPs

As versatile safeguarding polymeric platforms towards hazards, mass-produced MIPs are facing the challenge of complying with green sustainable development requirements. As mentioned, MIPs are smart materials with properties including reusability and chemical and mechanical stability, which are in line with green chemistry concepts [24]. However, in most cases, large quantities of MIPs based on the use of excess organic solvents and nonrenewable hazardous chemicals are produced. According to the 12 principles of green chemistry [25], focusing on reducing chemical inputs and efficiently processing by-products, some protocol steps required to obtain MIPs can pose a threat to both humans and the environment. Another relevant environmental impact of MIPs is the waste generated mainly due to unsuccessful imprints, requiring proper disposal. Nonetheless, the obtaining and using of advanced MIPs in various fields, including sensing and decontamination applications is imperative. 

Therefore, various strategies are followed to support green chemistry in MIP development: using bio-based monomers/solvents, designing multi-use and self-cleaning MIPs within a safe environment, neutralization of templates, and obtaining/recovering eco-friendly/biodegradable MIPs/their wastes [26,27,28]. Other green approaches involving smart imprinting strategies have emerged, such as dummy-template, multi-template, click chemistry, and stimuli-responsive imprinting (magnetic-, photo-, thermo-, pH- and dual/multi-responsive technology), while microwave-assisted heating techniques for MIPs have been less encountered [29]. Table 1 contains a systematic presentation of these imprinting strategies and their main characteristics. 

One of the most relevant greener aspects of MIPs is the use of bio-derived precursor materials, including green templates, fluorous or aqueous media, crosslinkers and initiators, functional monomers from renewable resources, and porogens/solvents [30]. Different types of biomass waste [31], i.e., chitosan, cellulose, activated carbon, carbon dots [32], cyclodextrins, and waste extracts were exploited; other bio-based monomers such as alkoxysilane, ionic liquids (ILs), metal ions such as Zn^2+^, biomolecules or self-polymerizable dopamine and vegetable oil-derived epoxidized soybean acrylate (ESOA) as cross-linker [20]. Thanks to their properties, relevant mentioning nonvolatility, miscibility and eco-friendliness, ILs are also used as a dummy template, a crosslinker, a porogen, or an additive. Other novel alternative solvents used to improve the green features of MIPs are deep eutectic solvents (DESs) [33].

**Table 1:** Recent smart imprinting strategies and their attributes, including bio-based aspects.

Imprinting Strategy	Characteristics	Refs.
**Dummy-template/Segment imprinting**	Replacing hazardous or very expensive targets with a dummy template that emulates the size, conformation, and functional groups of the target except for its undesirable characteristics, avoiding all types of risks and hazardous waste; using green dummy templates with high solubility to avoid poor solubility in porogen media; reducing danger to personnel; offering availability of using other analytes; segment/fragment imprinting (the case of biomacromolecules or hazardous templates) replacing dummy imprinting when using a partial target as a pseudotemplate for cost-effectiveness, regenerability, and non-toxicity.	[26,34]
**Dual/multi-template imprinting**	Versatility by the use of two or more target templates to generate multiple types of active sites in a single polymer material; comprising the use of self-synthetic and dual/multiple functional monomers and dual/multiple template ions; rarely reported for bio-macromolecules, affecting thus the heterogeneity of binding sites and poor selectivity; highly desirable for sustainability by simultaneously recognizing multi-templates; several different templates can be extracted, separated, and detected.	[29,35]
**Stimuli-responsive (SR)** **imprinting**	Smart polymers offer a plethora of alternatives for producing specific, powerful responses to a wide range of stimuli, i.e., changes in pH, gas, temperature, solvent, radiation, and biological or chemical agents; two main synthesizing methods: grafting/incorporating the SR into MIPs and the integration of SR elements within the MIP network; the use of safe biomaterials as SR will replace hazardous byproducts like ozone.	[26,36]
**Click chemistry** **imprinting**	Highly reliable one-pot synthesis tool first proposed in 2001 by Sharpless; biocompatible small-molecule reactions, generally used in bioconjugation with moderate reaction temperatures, leading to inoffensive byproducts; relies on new compounds and combinatorial libraries through heteroatom links (C–X–C).	[26,29]
**Microwave-assisted heating** **imprinting**	Widely applied to almost all types of polymerization based on their heating speed, selectivity, and efficiency properties; rapid energy transfer and high energy efficiency of microwave irradiation.	[28]

Besides the aforementioned smart imprinting strategies, Arabi and co-workers [26] proposed a greenified MIP map, available up to 2030, and revealed green and complementary alternatives for MIPs, including computational design strategies such as molecular dynamics and density functional theory [37], solvent-free and non-toxic metal-matrix imprinting, and avoiding post-imprinting [30]. Other greener synthetic ways imply ultrasound and magnetic field-assisted processes, miniaturized techniques (including micro SPE using pipette-tip, MIP-sol–gel in tablet form, or MIP-coated hollow fibers) especially in food and biological sectors, and supercritical fluid technology [38]. As current trends to design miniaturized and portable devices, with real-life applications and point-of-care testing, incorporating MIPs in microfluidic or biochips systems, nanosensors, and wearable sensors (optical and electrochemical) [39,40], have provided high selectivity and sensitivity. More recently, Marć and co-workers [41] proposed a dedicated tool and user-friendly software (termed AGREEMIP) with selected case studies for green assessment of MIP synthesis protocols. In this regard, AGREEMIP is based on an investigation of 12 criteria that correspond to the greenness of different reaction mixture constituents, energy requirements, and the details of MIP synthesis procedures; transformation of every criterion into a standardized 0−1 scale and weighted averaging of the criterion scores into an overall score, leading to interpretable pictogram with scores equal to 0 for nonacceptable situations and scores equal to 1 assigned to the greenest case, respectively.

## 2. MIPs Designed for CWAs and Other Hazardous-Related Compounds

In terms of public security and human safety, the detection or decontamination of chemical warfare agents (CWAs) is of great concern and interest to researchers worldwide. MIPs designed for CWAs or related compounds have a variety of purposes, including biomimetic enzyme catalysis, separation/extraction, clinical diagnosis, detection, and decontamination. Of these, decontamination and detection have mainly been reviewed.

**Figure 2:**
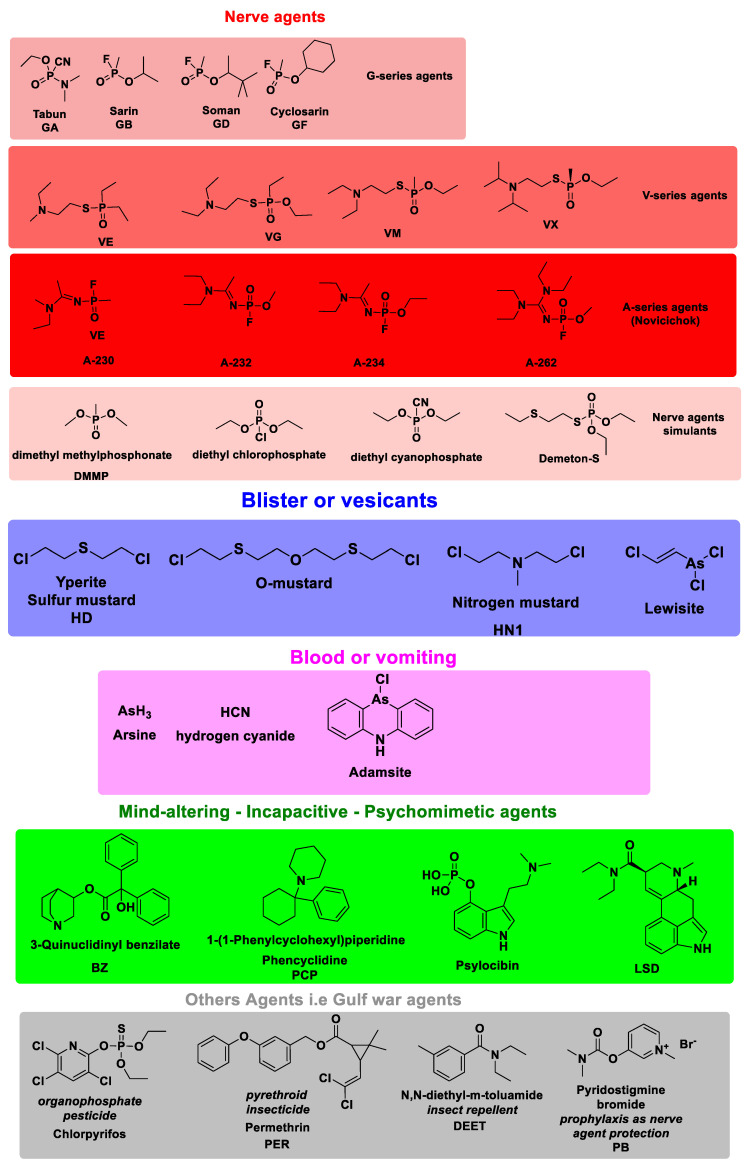
Classification of the main CWAs based on their chemical structure.

Since World War I and up to date, these highly toxic weapons have become a continued global risk and one of the crucial areas of research priorities in the fields of terrorism, public security, and human health. Although the CWAs and their degradation products have been prohibited by the Organization for Prohibition of Chemical Weapons (OPCW) [5], some CWAs are still being used in terrorist attacks or military operations and are widely stocked; the impact is illustrated by recent events in Germany (2020), Syria (2013, 2018—sarin attacks leading to approximately 1400 civilian deaths), VX in Malaysia in 2017, and other very recent relevant incidents related to the use of toxic chemicals acting as acetylcholinesterase inhibitor—namely Novichok agents, i.e., UK (2018) and the case of Alexei Navalny poisoning in 2020 [42,43]. The extensive and early efforts of OPCW to fulfill the CWC’s mandate to end the development, production, stockpiling, transfer, and use of chemical weapons led to the Nobel Prize for Peace in 2013 [5] (and 100% of the chemical weapons stockpiles verifiably destroyed declared by possessor States). Gas, liquid or aerosol, or powder forms **CWAs** are generally classified considering their chemical structure (Figure 2) as nerve agents and their simulants, blister or vesicants and stimulants, blood or vomiting agents, incapacitating (although a mind-altering agent, LSD as a psychomimetic compound will be included within illicit drugs section), other agents, i.e., Gulf war agents, and irritants/riot control agents [44]. It is worth mentioning that nerve agents belong to the chemical group of organophosphorus OP compounds (including pesticides), i.e., malathion, diazinon, chlorpyrifos, paraoxon, parathion, coumaphos, fenthion, fenitrothion, profenofos, phosmet, fenamiphos, triazophos. Since they significantly pose an environmental risk to human health, soil, and water, this section also comprises organophosphate pesticides (OPPs).

Though the OPCW database contains hundreds of CWAs, precursors, and degradation products, Table 2 comprises the most commonly encountered CWAs alongside their attributes (like toxicity, latency, persistency, or transmissibility) and effects during exposure, including inhalation, dermal absorption, oral ingestion, and/or injection [42,44]. In addition to early and specific decontamination, treatment, including some antidotes, is available for the listed hazardous agents (Table 2).

As mentioned above, the latest researched class of hazardous-related compounds is the highly lethal OP agents known as Novichok agents or A-series. In light of the recent events and considering the use and great interest of these new hazardous compounds since 2018, the Novichok agents were explicitly added to Schedule 1 of OPCW [45]. The Novichok class, as the 4th generation of highly lethal and persistent agents, was used by the URSS after the 1970s and lately grabbed worldwide attention after the above-mentioned incidents. According to Opravil and co-workers [46], five A-type agent structures known with unique amidine moiety (A-230, A-232, A-234, A-242, A-262), and another one is unknown, highly toxic as VX (toxicity mainly through dermal exposure), but more challenging to detect and easier to manufacture than VX. The stability and decontamination of these harmful compounds have been studied less due to the restrictive experimental date and unavailability; thus, effective countermeasures and methods of decontamination are very much needed. Until now, only three recent reports studying possible methods for the degradation of Novichok compounds (enzymatic, using MOFs or standard decontaminants) using the actual agents were explored [47,48,49].

To ensure homeland security and safety from the threats of CWAs via chemical analytical, food, and environmental applications, monitoring and novel emerging technologies and methods based on several types of sensing and decontamination are necessary.

### 2.1. Detection/Sensing of CWAs and Related Compounds

This subsection briefly overviews the main detection methods employed for organophosphate-based agents, including CWAs, pesticides, and simulants, using MIPs as versatile platforms via electrochemical and optical mechanisms. Due to their effect as an acetylcholinesterase (AChE) enzyme inhibitor, the organophosphate pesticides (OPPs), which are widely used in agriculture, are also included herein. Table 3 highlights an array of the latest trends for MIP development used for the detection of CWA and CWA-related compounds, and a comparison of their analytical performances. Other MIPs used as recognition elements for OPPs and environmental pollutants, together with their impact on human health and the environment, were recently discussed elsewhere [50].

In recent years, a wide range of analytical methodologies and detection methods has emerged to identify and recognize CWAs, including commonly analytical high-performance liquid chromatography HPLC, liquid chromatography-mass spectrometry LC-MS, gas chromatography-mass spectrometry GC-MS, capillary electrophoresis, and immunoassay tests [50]. However, these approaches face drawbacks such as harsh conditions, high cost, sophisticated knowledge requirements or procedures, non-portability, and lack of sensitivity and selectivity.

Thus, (ultra)sensitive and versatile optical detection techniques coupled with MIPs are highly recommended, especially in the field of biotherapeutics, food safety, and environmental monitoring applications. For this purpose, several optical techniques [72,73], such as UV–vis and infrared spectroscopy, surface-enhanced Raman spectroscopy (SERS) [74,75], surface plasmon resonance (SPR), and fluorescence quenching (using quantum dot QD, silver, platinum, and gold nanocomposites) [51,76], colorimetric and chemiluminescence (CL) [52,53,54], have been well integrated into MIP sensing platforms for CWAs detection. An example of a chemiluminescence biosensor for diazinon indirect detection was reported by Abdolmohammad-Zadeh and co-workers [53], using a molybdenum disulfide/zirconium MOF (MoS_2_@MIP-202(Zr)) nanocomposite; the sensing was based on the peroxidase mimic of the prepared nanocomposite on NaHCO_3_–H_2_O_2_chemiluminescence system as well as on the inhibitory effect of diazinon on the enzymatic activity of AChE. The reliability of this CL sensor was proved in real water samples with high recovery and a limit of quantification of 0.40 nmol L^−1^. Other studies use MIP-based techniques for CWA detection by quantifying the mechanical responses, such as quartz crystal microbalance (QCM) and surface acoustic wave (SAW) devices. For instance, Wang et al. [55] developed a SAW gas sensor based on the variations in the resonance frequency of piezoelectric crystals for dimethyl methylphosphonate (DMMP) sensing. Nonetheless, some of these methods are expensive or suffer from poor photostability.

Among the variety of tools developed for CWA detection, electrochemical sensors offer unrivaled merits of high sensitivity, specificity, and operational simplicity, proving to detect a broad spectrum of analytes in all phases, predominantly liquid and gaseous. For the fabrication of electrochemical MIP chemosensors, electropolymerization has been touted as an excellent procedure for polymer deposition onto transducers due to the precision with which the thickness of the layer can be controlled by time measurement vs. the applied current or potential [50]. Examples of such electrochemical MIP chemosensors for CWAs detection, with enhanced sensitivity, have been coupled to conductive polymers [56,57], single or multiple transition metal-based MXenes [58], multi-walled carbon nanotubes [59], metal nanoparticles [60], carbon quantum dots or oxides [61,62]. Moreover, photoresponsive MIP sensors based on poly (styrene-*co*-methyl acrylic acid) and carboxyl-capped polystyrene microspheres were reported by Chen et al. [77] and used for detecting profenofos in tomatoes and mangosteen. Within a linear range of 0–15 μM profenofos in standard samples, the MIP sensor registered quite good recovery rates (94.4–102.4%).

Recent insights into novel fabrication methodologies and electrochemical techniques have resulted in new generations of nano-sized modified electrochemical sensors (for example, when using MOF-74 [63,64], able to address many of the limitations of conventional methodologies. Electrochemiluminescence (ECL) has attracted much attention, as well, due to the synergies between electrochemistry and chemiluminescence, which lead to high selectivity and sensitivity as well as superior controllability and low background noise [72]. Such ECL sensors were recently developed for the ultra-sensitive detections of chlorpyrifos [65] and malathion [66].

Sezigen and co-workers [67] developed an electrochemical sensor utilizing the MIP based on 4-aminobenzoic acid (4-ABA) and tetraethyl orthosilicate for the detection of nerve agent VX metabolite ethyl methylphosphonic acid (EMPA) in human plasma and urine samples, with imprinting factors over 3. Alongside the low LOD up to picomolar values in standard, plasma, and urine solutions, this work provided a selectivity study that included isopropyl methyl phosphonic acid IMPA-a major hydrolysis metabolite of nerve agent sarin, n-benzylphosphonic acid n-BPA, and MPA as interferents. Thus, this sensor exhibited distinct recognition and binding properties toward EMPA molecules and a range of exceptional features, proving a promising strategy for the detection of other biomarkers like sarin, soman, or Novichok in human biological samples. Another example of the detection of nerve agent stimulant parathion in different matrix environments comes from Yağmuroğlu [68], who developed an MIP-based potentiometric sensor using N-methacryloyl-L-serine monomer (MA-Serine) and carbon paste for preparing the recognition element; in this case, the response time of the sensor (20 s) was quite low. 

Lately, point-of-care testing (POCT) systems have attracted much attention because the necessity of laboratory staff and facilities is cut out, while the method implies the miniaturization of the diagnostic tools and rapid testing cycles as well as the use of low amounts of reagents. In this respect, recent papers [69,70,71] report the detection of thiodiglycol (TDG), sulfur mustard poisoning metabolic marker), using molecularly imprinted test strips and POCT. The same group of Luo and co-workers (i) designed a MIPs-based lateral flow assay (MLFA), a commonly used POCT method, using AuNPs that could bind TDG through Au–S interaction loaded on a conjugate pad [69]; (ii) assembled MIPs on the surface of ethylene imine polymer (PEI)/poly(vinyl alcohol) (PVA) electrospun nanofiber membranes, using concentrated AuNPs as the signal reporting units [70]; (iii) developed a test strip based on a MI sensitive membrane, by coating MIPs on a nitrocellulose membrane; when the sample contained TDG, the MIPs could specifically bind TDG. The analytical performances of the POCT sensors for TDG detection are listed in Table 3.

### 2.2. Decontamination of CWAs and Related Compounds

To date, it is urgent to discover practical and efficacious artificial agents for the decontamination or detoxification of CWAs. While the management of toxicity prioritizes early decontamination to reduce sequelae and further contamination, the chemical reaction method exhibits the great advantage of permanent detoxification. The literature shows that most of the commonly used disinfectants possess low efficiency, difficulties in military procedures, strong corrosivity, storage difficulties over time, and severe environmental pollution. According to the new findings, only a few reports of the CWA's decontamination methods combined with MIPs' performances for effective high-performance protective materials against hazardous agents were found. 

Wang and co-workers proposed a self-detoxifying method for organophosphates utilizing MIP electrospun fiber scaffolds and spectroscopic methods [78]. An amidoxime-based functional polymer (PMAOX) containing amidoxime groups that can act as nucleophiles and ligands for the formation of catalytic active sites was blended with commercial polyacrylonitrile (PAN) to obtain the blend fiber material via electrospinning. MI was carried out upon the fiber surface incorporating diethyl(4-nitrobenzyl) phosphonate (D4NP) template-coordinating assemblies and crosslinking of the side chain. The MIP electrospun nanofibers MIF-Zn-PMAOX/PAN-6/4 catalyze the degradation of paraoxon with a half-life of 32 min, and MIF-Ag-PMAOX/PAN catalyzes the degradation of parathion with a half-life of 18 min. Other performances of the MIF-Zn-PMAOX/PAN-6/4: the apparent reaction rate constant (*k*_obs_) catalytic degradation of paraoxon is 7.7 × 10^−3^ min^−1^, *t*_1/2_ = 90 min, and *k*_obs_ of parathion is 7.1 × 10^−3^ min^−1^, *t*^1/2^ = 98 min. 

A relevant study demonstrated a broad-spectrum treatment option for OP cholinesterase inhibitors, which directly countered the effect of the poison *malathion*, a common pesticide, without attempting to dislodge it. The restoration of acetylcholinesterase activity was measured by the administration of functional MIP nanoparticles, prepared using peptides epitopes. Enzymatic acceleration of the cholinesterase was observed at 162 ± 17%, the rate of erythrocyte ghosts without bound nanoparticles [79].

In the same perspective, Disley et al. [80] successfully manufactured an MIP from chitosan with the ability to selectivity trap DMMP, a gas mimic for sarin and soman. The 1:3 ratio of DMMP to chitosan was the optimum ratio to produce the most energetically stable template−monomer complex, calculated by density functional theory having the B3LYP/6-31G level. The MIP trapped 4554 (±227) ppm (4.55 mg/g) DMMP and outperformed both the NIP (156 (±20) ppm (0.16 mg/g)) and activated charcoal (82 (±17) ppm (0.08 mg/g) and were obtained by GC-MS; thus, the DMMP-responsive MIP can be an ideal absorbent material in gas masks or gas filters. However, the MIP was not reusable due to methanol extraction procedures.

Inspired by the highly active catalytic performances of MOFs towards CWAs, another recent study by Jiang et al. [81] brings the design of a MIP/MOF hybrid catalyst hydrogel by integrating enzyme-mimic MIP with Zr-MOF UiO-66-NH_2_. Specifically, hydrogel polymeric matrix MIP-pyridyl-amidoxime functional monomer (PAAO-SA) with self-buffering and hygroscopic capability was prepared. The half-life of liquid phase hydrolysis catalyzed by MIP/UiO-66-NH2-0.5 achieves 9.4 min for diethyl-4-nitrophenyl phosphate (DENP) and 10 min for dimethyl 4-nitrophenyl phosphate (DMNP), and solid-state hydrolysis present rapid degradation efficiency with a half-life of 28.6 min. In addition, composite catalyst MIP-Ag/UiO-66-NH2-0.5 that retains substrate selectivity of MIP enables the simultaneous degradation of two types of organophosphorus substrates (phosphate and thiophosphate) by MIP and MOF in a synergistic way. Hence, Zr-based metal-organic frameworks (Zr-MOF) have demonstrated effective detoxification against toxic chemicals of OP and outperformed in exceptional porosity, high surface area, and amenability to modular design. MIP catalysts are well known for the accurate designing of the active sites and have been widely used as mimics of natural enzymes with enzyme-like catalytic reactivity but higher stability.

Another strategy worth mentioning that can efficiently catalyze the degradation of OP toxicants relies on the preparation of enzyme-like MIP double network hydrogel by integrating the catalytic active polymer and the cationic polymer polyethyleneimine (PEI). In this work, Wang et al. [82] engineered MIP-DN hydrogels that exhibited a half-life of catalytic degradation of paraoxon in pure water of only 26 min. Also, as extensively used in sensing applications, cobalt and MIPs are used in the detection of carbon monoxide and decontamination of nerve agents, utilizing porous sol–gel materials [83].

## 3. MIPs Designed for Explosives Assessment

The analysis of terrorist attacks in the last two decades reveals a constant increase in the number of events and a persistent shift towards “soft-targets” (simple public and private buildings, targeting and killing individuals, typically civilians), which are perpetrated through the use of explosives [84]. In response to this, applications connected to security and counterterrorism have made the detection of explosives and chemicals related to explosives high priorities [85,86,87,88]. Thus, significant research efforts have been directed towards novel designs for detection as well as for the improvement of existing methods. The research direction targeted new concepts/materials [89,90] for explosive sensing that can permit field deployment, portability, miniaturization, high-throughput detection [91], and stand-off measurements while assuring selectivity to eliminate false reports [85,92,93,94]. Explosive trace detection methods include gas chromatography (GC), high-pressure liquid chromatography (HPLC), ion mobility spectroscopy (IMS), mass spectrometry, infrared absorption spectroscopy, and Raman scattering, which are not all easily applied in the field, and all can benefit from methods for sample collection facilitation or selectivity improvement [93]. To accurately and selectively detect explosives, MIPs facilitate the identification/selective retention process, which is essential. Thus, through careful MIP design and synthesis, polymeric structures with tailor-made gaps or areas that are sized, shaped, and functionally matched to target/explosives molecules are obtained. 

**Figure 3:**
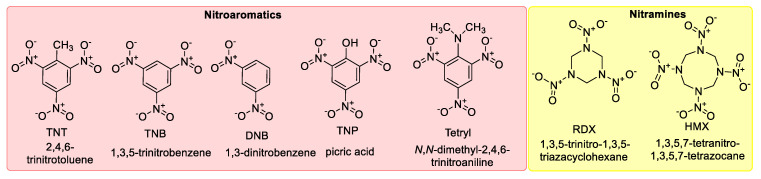

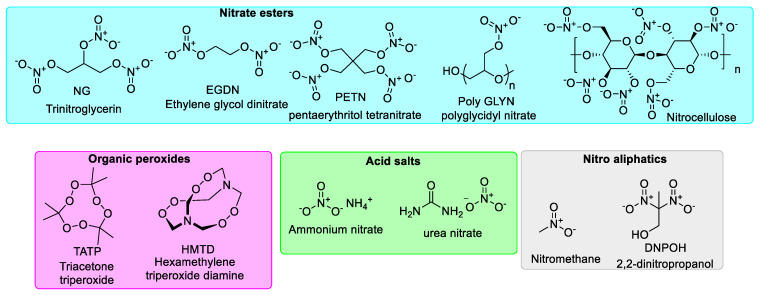
Classification of explosives based on their chemical structure.

Explosives come in a variety of forms, and they can be divided into several groups based on their chemical structure (Figure 3). Nitroaromatic explosives, which are found in ammunition, have the potential to contaminate soil and groundwater [95]; they include examples such as TNT and TNP. High vapor pressure di-nitrotoluenes are found in blasting gelatin formulations, in gun/rocket propellants, and as contaminants in TNT manufacturing. Propellants, detonators, and plastic explosives are among the military uses for nitrate esters, many of which are liquids. Furthermore, plastic explosives also contain brisant explosives like HMX and RDX [96]. Organic peroxides that are sensitive to heat, shock, and friction are frequently utilized in homemade explosives for improvised explosive devices [97]. In addition to being employed as a blasting explosive, ammonium nitrate/fuel oil (ANFO) is also frequently utilized as fertilizer. ANFO and urea nitrate have been utilized in many improvised explosive devices [98], where aliphatic nitrates and fuel combinations can also be found [99].

The modern detection process entails the obtaining of an indication/change/alarm caused by the presence of the target molecule or target-related species. This can be attained commonly in the case of explosives by one of the following methods: (i) optical sensors, (ii) electrochemical sensors, (iii) mass sensors, or (iv) biosensors [100,101] as for any other use in the case of MIPs designed for explosives their advantage resides in the molecular design or imprinting specificity. Thus, multiple monomers can be utilized and tailored to establish interactions with the targeted explosive molecules, especially by exploiting the interactions with nitro groups, while the crosslinking density can also affect the selectivity. In addition, MIPs can constitute a facile, reliable, low-cost solution with low detection limits suitable for field deployment in applications such as environmental control or security enforcement.

Detection of explosive traces is a complex undertaking. In security applications, sampling wands are used for particulate sampling. Therefore, the modality of sampling is critical and also involves a degree of training/intuition for the personnel performing the collection to identify/select the areas with a high probability of encountering explosive traces. Additionally, vapor sampling is highly difficult due to the very low vapor pressures of many explosives [102]. Also, in the case of liquid samples, the wetting characteristics of the materials must be well-controlled to permit efficient sampling. The operational environment and parameters are also important for explosive trace detection, requiring fast analysis, reduced number of false alarms, and efficient, streamlined workup procedures. One of the most important aspects is, therefore, selectivity, which ensures a low number of false alarms. New technologies should be carefully designed to ensure selectivity, sensitivity, and operational and financial requirements.

Molecularly imprinted polymers (MIPs) possess specific binding sites within their polymer matrix, enabling precise, efficient, and selective detection of explosives molecules. These capabilities are primarily harnessed through electrochemical or optical methods, facilitating the accurate identification of explosive analytes. Some of the most recent publications describing MIPs for explosive detection are listed in Table 4.

The following subsections illustrate specific attributes of MIPs designed for explosives sensing applications that rely on either optical or electrochemical detection principles.

### 3.1. MIPs for Sensing/Detection of Explosives by Optical Techniques (e.g., Colorimetry, Fluorescence, Surface-Enhanced RAMAN Scattering (SERS)) 

The development of optical sensing technologies for explosive trace detection employs concepts such as absorption, reflection, fluorescence, and RAMAN scattering to recognize chemical fingerprints related to the presence of explosive traces [87].

MIP-based optical sensing can capitalize on the binding affinity of the MIPs and or the optoelectronic characteristics resulting from the careful selection/design of the monomers used for the construction of the MIPs polymeric architecture [108,109]. The essential requirements for optoelectronic MIPs are photochemical and thermal stability, as well as the capacity to offer an optical characteristic modification proportional to the target analyte with high selectivity.

**Figure 4:**
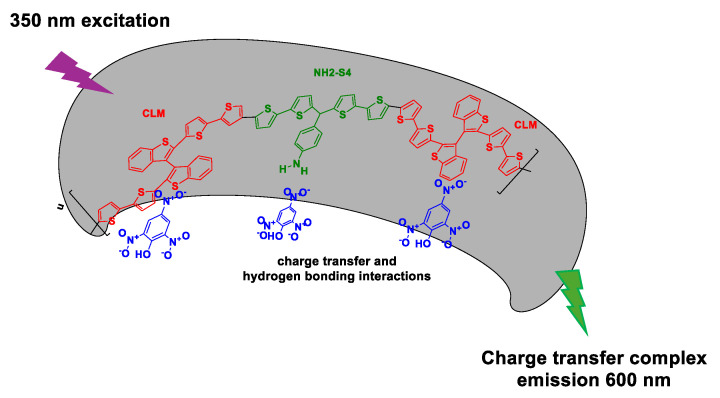
MIP structure and interaction for picric acid detection, adapted from Huynh et al. [110].

Fluorescence-based methods are some of the most preferred approaches for nitroaromatics trace detection due to their high sensitivity, good portability, the possibility for eco-friendly design, and simple utilization protocols. Since many nitroaromatics are non-fluorescent, detection performance is enhanced by using fluorescent sensory elements. The intensity (quenching or amplification), wavelength, anisotropy, or lifespan of the fluorescence changes in correlation with the concentration of the explosive target molecule and exposure duration. One of the important steps in designing new solutions for explosive traces detection based on fluorescence entails elucidation of the interaction mechanism between the fluorescent sensing materials and target molecules. The interaction can entail photoinduced electron transfer, resonance energy transfer, electron transfer, or intramolecular charge transfer processes [109]. The design of fluorophores with adequate response has received significant research attention, with examples including small molecules, nanoparticles (quantum dots), and conjugated polymers, which offer an effective electronic communication pathway between the quencher along the polymer chain. In this context, Huynh et al. [110] developed a chemosensor for picric acid determination using an imprinted polymer involving a recognition unit (NH2-S4) cross-linked to a fluorophore transducer (CLM) (Figure 4). The MIP-picric acid film was demonstrated to be a good chemosensor candidate material due to its emission in the visible range and excellent picric acid limit of detection (at the sub-nanogram per liter picric acid concentration, at least 0.2 ng/L picric acid). Additionally, the CLM component played a multiple role as a functional and cross-linking monomer as well as the fluorophore in this chemosensor. Thus, the CLM assures the stability of the MIP (crosslinking) the functional monomer with the formation of MIP cavities, while its fluorescence property allows signal registration and quantification [110].

The photostability problems of small fluorescent molecules can be addressed by using nanoparticles (quantum dots) that have been integrated within molecularly imprinted polymers [89]. For example, Xu et al. [111] have developed an ultrafast fluorescent probe based on molecularly imprinted polydopamine-coated silica-CdTe particles for visual p-nitrophenol monitoring from aqueous samples. The developed materials permitted the visual detection (by the naked eye) of p-nitrophenol within 2 min through the exploitation of a fluorescence quenching mechanism [111], while the limit of detection was 56.68 nM. Another appealing alternative for fluorescence quenching-based gas sensors that were explored for explosives and taggant molecules involved perovskite nanocrystals integrated into an MIP nanocomposite design [103]. In a recent report, Aznar-Gadea et al. [103] employed CsPbBr_3_ nanocrystals embedded in a polycaprolactone matrix and developed a rapid sensor for 3-nitrotoluene (limit of detection 0.218 μg mL^−1^)with a rapid detection in less than 3 s.

Surface-enhanced Raman scattering (SERS) has also received significant research attention as a strategy for explosives detection [112], and it can also be integrated into MIP design. Recently, Aznar-Gadea et al. [113] reported a MIP—silver nanoparticles composite as a selective and sensitive platform to detect and quantify 3-nitrotoluene (used as a taggant molecule). The sensor is based on Ag NPs embedded inside the polyethyleneimine (PEI) as a polymer matrix and 3-nitrotoluene as a template to generate specific molecular recognition sites. The reported synthesis of the Ag NPs and the molecular imprinting process take place simultaneously during the baking step. Thus, the obtained nanocomposite combines the optical properties of the silver nanoparticles with the specific characteristics of the MIPs. However, these sensors present a slow response time, and, as a consequence, their application would be limited to some specific fields. In another example of SERS and MIP application, Liu et al. developed SERS in combination with microfluidics to obtain a limit of detection for trinitrotoluene (TNT) and 3-nitro-1,2,4-triazol-5-one (NTO) in the order of 10^−7^ and 10^−8^ M, respectively [91].

Furthermore, using the MI technique and various polymerization procedures, MIPs in different shapes and sizes for explosive detection were developed, e.g., membranes [114] or thin films [115]. An attractive approach to obtain MIP-sensitive layers for TNT recognition is the wet phase inversion method (a two-pot post-polymerization imprinting procedure), which was used by Lazau and coworkers [114]. For this, the hydrothermal method and the MI were combined to develop an efficient and reusable bicomponent element with a stratified structure, a.k.a. thin oxide film (TiO_2_) grown in situ onto FTO substrate and functionalized with 2,4,6-trinitrotoluene (TNT)-molecularly imprinted polymer (MIP) membrane, as a proof-of-concept in developing a TNT portable and reusable sensor; the poly (acrylic acid-co-acrylonitrile) film exhibited good properties, especially in terms of reusability. Further studies of Gavrila et al. [115], dealt with the development of a multilayered capacitive quartz-Cr-Au-TiO_2_-MIPsensor, tested in vapor state, with fast response times (less than 25 s), low sensitivity to humidity (the variance of capacitance from 5% to 31% humidity was around 5.6 pF), and high specificity for TNT (IF = 1.9) and selectivity vs. dinitrobenzene (DNB). This study describes the auto-assembly mechanism of TNT with functional diamino-silanes (i.e., N-(2-aminoethyl)-3-aminopropyl methyl dimethoxysilane), via “double” Meisenheimer complexes, in the form of 1.53:0.88 = TNT:1141-D (M/M), which were further condensed by sol-gel to yield a TNT-MIP film.

Future developments in MIPs using optical modification may target their design for field deployment, fast/accurate response, and reusability of the materials.

### 3.2. MIPs for Sensing/Detection of Explosives by Electrochemical Methods

The advantages of electrochemical analysis over spectral approaches include low cost, high sensitivity, and simplicity. In the field of nitro explosives detection, the molecular imprinting process has been combined with electrochemical sensor technology [116]. Selectivity, stability, cost-effectiveness, real-time monitoring, and miniaturization potential are just a few of the benefits that MIPs offer in the electrochemical detection of explosive analytes. While MIPs offer several advantages, they also exhibit limitations, including restricted conductivity and electrocatalytic activity, which sometimes lead to reduced sensitivity. Consequently, to enhance MIP conductivity, researchers have explored the integration of diverse nanomaterials, such as quantum dots (QDs), metal-organic frameworks (MOFs), Au, Pt, Ni nanoparticles/nanowires, and Pt/Au and Au/Ag bimetallic nanoparticles, etc. [117].

In this context, an interesting example of MIPs based on electroconductive polymers was developed by Sağlam et al. [105] consisting of glassy carbon (GC) electrodes coated with polycarbazole (PCz) films decorated with gold nanoparticles (AuNPs) by cyclic voltammetry (CV), to serve as MIPs for triacetone triperoxide (TATP) and hexamethylene triperoxide diamine (HMTD) sensing, within 0.1−1.0 mg/L, with a LOD of 15 μg/L for TATP and HMTD. Electroactive compounds such as paracetamol, caffeine, acetylsalicylic acid, aspartame, D-glucose, and detergent (including perborate and percarbonate) utilized as camouflage materials for peroxide-based explosives did not interfere with the suggested MIP electrochemical sensing approach [105].

Another example of electroconductive polymers MIPs was developed by Zheng et al. [107], including a selective electrochemical sensor for multiple nitroaromatic explosives detection based on poly (3,4-ethylenedioxythiophene)/laser-induced graphene-based MIP by utilizing trimesic acid (TMA) as the dummy-template and 3,4-ethylenedioxythiophene (EDOT) as functional monomer. Benefiting from the association of TMA and EDOT, the versatile MIP demonstrated excellent selectivity and sensitivity for representative nitroaromatic explosives, 2,4,6-trinitrotoluene (TNT), 2,4,6-trinitrophenol (TNP), 2,4-dinitrotoluene (DNT), 1,3,5-trinitrobenzene (TNB), 2,4-dinitrophenol (DNP), and 1,3-dinitrobenzene (DNB), with limits of detection of 1.95 ppb, 3.06 ppb, 2.49 ppb, 1.67 ppb, 1.94 ppb, and 4.56 ppb, respectively. Four common explosives RDX (hexogen), NG (nitroglycerine), PETN (penthrite), HMX (octogen) and other common compounds like urea, inorganic salt ions (K^+^, NO_3_^−^, SO_4_^−^), heavy metal ions (Pb^2+^, Cd^2+^), or urea were investigated for their interference with TNT detection, which was not significantly affected by common environment chemicals, indicating the exceptional selectivity of the developed materials [107].

In an example that shows that the imprinting process can fail, Głosz et al. [106] developed polycarbazole MIPs deposited on platinum (Pt) and glassy carbon electrodes and investigated their performance in detecting picric acid. According to quantum mechanical calculations, picric acid interacts strongly (31.43 kJ/mol) with the repeating units of polycarbazole, which is usually sufficient to achieve a significant improvement in sensor sensitivity. Nevertheless, the performance of the MIP layers was inferior to that of the unmodified Pt or GC electrodes, probably due to the relatively lower conductivity of the conjugated polymer layers in comparison with either Pt or glassy carbon electrodes.

In the search for sensitivity enhancement, Tancharoen et al. [118] developed a co-imprinting technique using a dinitrotoluene-dengue virus template for the elaboration of electrochemical sensors. The introduction of the dengue virus during the imprinting process was found to produce higher sensitivity to TNT than conventional imprinting. Furthermore, the largest margin of separation between signal responses to DNT, TNT, and PETN was also observed from the MIP sensor co-imprinted with DNT. The differences in simulated binding energy and conformation of DNT and TNT at the virus envelope protein might explain these differences in sensing performance [118].

In another study, Huynh et al. [119] employed thiophene polymers for MIPs development for several nitroaromatics 2,4,6-trinitrophenol (TNP), 2,4,6-trinitrotoluene (TNT), 1,3,5-trinitrobenzene (TNB), and 2,4-dinitrotoluene (DNT) and developed a simultaneous chronoamperometry and piezoelectric microgravimetry determination procedure. The limit of detection of the fabricated materials ranged between 0.27 to 0.69 and 0.02 to 0.76 μM for the chronoamperometry and piezoelectric microgravimetry, respectively, with the detectability of the piezoelectric microgravimetry lower than that of the chronoamperometry approach [119].

Based on the literature survey on MIPs for explosives sensing/detection by electrochemical techniques, it can be concluded that while MIPs improve the selectivity of nanosensors, the incorporated nanomaterials improve the sensitivity of MIP-based electrochemical sensors.

## 4. MIPs Designed for Illicit Drugs Assessment

The last two decades of increasing substance abuse have had a significant global impact, threatening people’s health, safety, communities, and the environment worldwide. Considering their major effects and origin, the main classes of **illicit** or prohibited drugs [120] include **psychoactive substances**, divided into different groups: **(i)** from naturally occurring/legitimate pharmaceuticals **narcotics** *morphine and codeine*-from opium poppy, to **synthetic narcotics (opioids)** such as *oxycodone*, *heroin*, *methadone*, *dextromethorphan*, *dextrorphan*, *noroxycodone*, *pentazocine*, *norpethidine*, and highly lethal **synthetic opioids** like *fentanyl*, with effects on the µ- and ƙ-opioid receptors and decrease/inhibit pain by inhibiting release of neurotransmitters), **(ii) stimulants** (*cocaine*, from the coca plant, *amphetamines, and precursors ephedrine/pseudo-ephedrine*, *semi-synthetic LSD or d-Lysergic acid diethylamide, MDMA or Ecstasy*, affecting the central nervous system to increase the feeling of excitement and diminishing tiredness), **(iii) hallucinogens/cannabinoids**, causing hallucinations and euphoria: naturally occurring *marijuana* (*cannabis*, from *cannabis sativa*), *mescaline*, from Peyote cactus, *psilocybin/psilocin*, from mushrooms; **(iv) depressants** (*sedatives/hypnotics/barbiturates*). Other prohibited drugs are doping ones, including **anabolic steroids** (such as *AKA*, *anabolic*–*androgenic steroids AAS*, *Deca-DurabolinND, Testosterone decanoate TS*, altering the protein synthesis within skeletal muscles and overall strength) and **peptide hormones** as performance-enhancing anabolic substances (*human growth hormone GH*, *erythropoietin EPO*, *insulin-like growth factors IGF-1*). 

**Figure 5:**
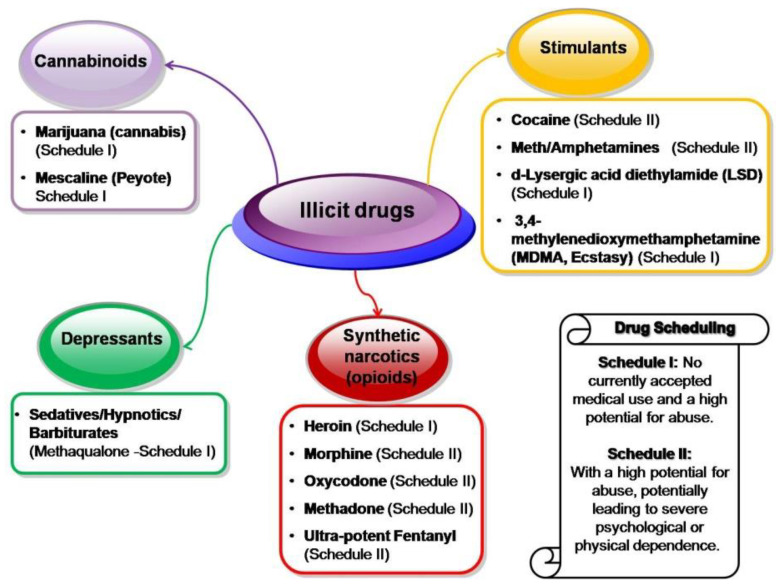
Classification chart of the main illicit drugs, depending upon the drug’s major effects and origin, as well the existent Drug Scheduling on the most hazardous (Schedule I and II, according to US Drug Enforcement Administration).

Yet, this review section highlights the main illicit drugs or “hard” drugs exclusively [121] (Figure 5), excluding the licit ones (i.e., antidepressants, alcohol, nicotine, benzodiazepines) and the doping drugs, with a focus on newly **ultra-potent synthetic opioids** (both Pharmaceutical-based agents such as Fentanyl or Fentanyl-derived products such as alfentanil, sufentanil, remifentanil and non-pharmaceutical fentanyl including acetylfentanyl, carfentanil, ocfentanil, and furanylfentanyl).

According to the World Drug Report 2024 launched by the UN Office on Drugs and Crime (UNODC) [122], there are 292 million drug users in 2022, a 20% increased number over 10 years. Globally, cannabis remains the most widely used drug (228 million users), followed by opioids (60 million users), amphetamines and cocaine (30 and 23 million users, respectively), and ecstasy (20 million users). The new trends mentioned by the European Drug Report 2024 are prevalent in Europe, including polydrug consumption (the use of two or more psychoactive substances, licit or illicit, simultaneously or sequentially) such as Ketamine (with rapid antidepressant effects and added to other drugs mixtures, including ecstasy powders and tablets, or ‘pink cocaine’) [123]. Synthetic cannabinoids (SCs) are a broad class of illicit drugs designed to mimic the effect of Δ^9^-tetrahydrocannabinol (THC, as the main psychoactive ingredient of marijuana), with short and long-term pathophysiological effects upon the endocannabinoid system [124]. Comprising a core, a secondary structure, and a linker with a tail group attached, SCs are generally classified after their aromatic core structure i.e., indole, indazole. Becoming commercially available or ‘legal’ alternatives to cannabis in some EU Countries, the most commonly encountered semi-synthetic cannabinoids are hexahydrocannabinol (HHC) and, more recently, hexahydrocannabiphorol (HHC-P) and tetrahydrocannabiphorol (THCP). Cocaine, amphetamine, methamphetamine, ecstasy or 3,4-methylenedioxymethamphetamine (MDMA), and more recently, compounds like cathinone are a group of synthetic central nervous system stimulants used to treat several diseases, including narcolepsy, depression, weight control and various psychological or emotional disorders [125]; however, nowadays, these stimulants are used for fun purposes, due to their ability to induce high energy, reduce appetite, or enhanced confidence effects. Thus, cocaine and related psychotropic compounds are available in the black market, although a record of more than 300 tons of cocaine was seized by EU Member States for the sixth year running [123].

### 4.1. Ultra-Potent Synthetic Opioids 

The term “opioids” refers to both the natural compounds called opiates (e.g., morphine, codeine), which are extracted from the opium poppy plant (*Papaver somniferum*), and their semi-synthetic (heroin, oxymorphone, oxycodone, hydrocodone) and synthetic derivatives (methadone, fentanyl, and its analogs) [126]. According to recent studies and news, synthetic opioids already represent a foreseeable crisis worldwide, particularly in the United States and Canada [127]. Beginning with the North American market and spreading across Europe, strong opioids like **fentanyl** and novel **nitazenes** have become much more prevalent, considering that they are more potent than heroin and most likely mixed into heroin. A new generation of synthetic opioids available on the global drugs market emerged: non-fentanyl “U” compounds like U-48800, U-49900, U-47700 and U-47931E or bromadoline), U-50488, AH-7921, MT-45. These new ultrapotent synthetic substances are known to be added to street drugs, ingested accidentally or used in terrorist attacks and rapidly produced in so-called homegrown laboratories using legal and easily cheap available precursors.

As published by a recent statistics report [128], drug overdose deaths involving opioids and heroin have also risen at an unprecedented rate in the past 20 years (in 2022 a number of 107,941, and in 2023, 74,808, only illicitly manufactured fentanyl and fentanyl-analogs deaths occurred in the U.S.; however, the overall rate of drug overdose deaths remained stable between 2021 and 2022). It is relevant to mention that the above-mentioned opioids are also sometimes seen in fake opioid tablets (like oxycodone blue or yellow tablets) and even occasionally in non-opioid drugs, like cocaine, benzodiazepines, and synthetic cannabinoids (SCRAs). These hazardous compounds act on the same targets in the brain as natural opioids (e.g., morphine and codeine) to produce analgesic (pain relief) effects or euphoria, confusion, dizziness, nausea, vomiting, urinary retention, and respiratory depression (within minutes of exposure). At low doses, they can act as painkillers and sedatives, although they can cause death at higher doses. 

Among the existent opioids trafficked on the West Coast in the 1980s, fentanyl [N-phenyl-N-[1-(2-phenylethyl) piperidin-4-yl] propanamide] stands out as one of the most popular and super-strong lipophilic opioids with over 500 related analogs of varying potency. As related by some new reports [129], carfentanil in powder, tablets, and liquid forms, is approximately 1000 times more potent than morphine (or 100 times more than fentanyl), leading to overdoses and overdose-related deaths, even among opioid-tolerant users (U.S., Canada and some European countries—very recently in Estonia, Ireland, France, Latvia). As used as a tranquilizing agent for large animals, carfentanil can be lethal in the 2 milligram range, depending on the route of administration and other factors. Non-fentanyl-derived compounds that are several times more potent than fentanyl are benzimidazole-opioids ornitazenes (such as isotonitazene, metonitazene, N-pyrrolidino-etonitazene, protonitazene, N-desethyl etonitazene)**,** increasingly detected in Canada and the USA are not well characterized [130]. Still, in 2024, etodesnitazene, N-pyrrolidino etonitazene, and protonitazene are permanently placed by the United States government in Schedule I of the Single Convention [131], alongside temporarily voted butonitazene, flunitazene, isotonitazene, and metodesnitazene in 2022.

Considering the toxicity of carfentanil, fentanyl-related, or nitazenes and the fact that they act as mu-opioid receptor agonists, only properly trained and outfitted law enforcement professionals should carefully follow safety protocols to avoid accidental exposure. Extensive efforts via guidance or mitigate solutions to deal with these hazardous opioids have been made by authorities from different continents, as addressed by, for example, UK government guidelines [132]. Nevertheless, new therapies for opioid overdose are urgently needed. One of the first-line antidotes for the reversal of opioid (such as fentanyl and morphine) overdose is the opioid antagonist naloxone (NLX, Narcan^®^) [133]. NLX works by binding to the brain’s opioid receptors, thereby kicking fentanyl off those receptors and blocking its effect on the body in the process. However, naloxone cannot be administered prophylactically, and its short duration of action demands multiple administrations, especially for highly potent carfentanil (which binds to the receptors more strongly than fentanyl). NLX is also available in combination with a semisynthetic compound from the opium category buprenorphine (BUP), which can be extracted using MIPs. Also, immunotherapeutic strategies, including antibodies, have been explored to counteract the effects of synthetic opioids. Another promising medication for reversing fentanyl and analog-induced overdose is the human monoclonal antibody CSX-1004 with picomolar affinity, producing a 15-fold potency reduction in respiratory depression [134].

To find effective alternative treatments for opioid overdoses and traditional toxicology screenings to detect these compounds at such low doses and in real samples, cost-effective on-site sensing devices are essential. Screening for illicit drugs is usually performed by a combination of gas or liquid chromatography (GC/LC) and mass spectrometry (MS), leading to high sensitivity and selectivity [135,136,137]; however, most of these reported methods require expensive equipment, high organic solvent consumption, and low portability, and are time-consuming. To cover these limitations, the preparation of molecularly imprinted polymers (MIPs) and metal-organic frameworks (MOFs) can be suitable solutions to overcome the above-mentioned drawbacks. More, many efforts have been made to miniaturize classical extraction methods and reduce the solvents, including solid phase microextraction (SPME). The SPME with GC-MS or HPLC was employed to detect narcotic substances in forensic medicine laboratories, such as amphetamine, cocaine, and its derivatives [138,139,140]. As shown in the research of Kardani et al. [139], a synthesized monolithic fiber of MOFs MIL-Al (53)-deep eutectic solvent (DES)/MIP combined with SPME was used to determine amphetamine derivatives and modafinil from unauthorized medicinal supplements, leading to the following sensor performances: LOD in the ranges of 0.023–0.033 μg L^−1^ and recoveries ranging from 95.14 to 104.63%. Fu and coworkers [141] prepared an MIP as a sorbent using ephedrine or pseudoephedrine as a dummy template; SPE followed by LC-HRMS for the enantiomeric determination of five cathinones in river water samples were applied. Under optimal conditions, the method developed provided relatively satisfactory recoveries ranging from 67.6 to 83.2%, and LOD ranged from 0.3 to 0.8 ng/L. Another paper on the extraction of five synthetic cathinone SCs (cathinone, methcathinone, mephedrone, methylone, and ethylone) from complex matrices using dummy MIPs via bulk polymerization is reported by Han and coworkers [142]. The established SPE combined with HPLC-MS/MS method showed low limits of detection (0.002–0.1 ng mL^−1^) and good recoveries (84.1–97.7%) in wastewater, urine, and cocktail samples. In a similar study done by the same group [143], five amphetamine-type stimulants ATSs (amphetamine, methamphetamine, 3,4-methylenedioxyamphetamine, 3,4-methylenedioxymethamphetamine, and 3,4-methylenedioxy-N-ethylamphetamine) were determined using dummy MIP–agarose gel mixed matrix membrane from wastewater and urine samples; the HPLC-MS/MS method exhibited low LOD (0.1~3.0 ng L^−1^) and recoveries in spiked urine of 82.3~95.7%. Another selective determination of methamphetamine in forensic sciences was performed by Brito and coworkers [144] using MIP-assisted paper spray ionization mass spectrometry (MIP-PSI-MS). Shortly, the MIP was synthesized on the surface of a paper, producing a chemically selective paper surface with molecular recognition sites for the drug. Methamphetamine was detected at higher ion signals compared to other drugs, such as lysergic acid diethylamide (LSD) and cocaine, leading to a LOD of 0.8 μg L^−1^ and recoveries close to 100%. Based on the few data reported [145], we have provided the newest detailed overview of the attractive properties of real-time electrochemical and optical sensors incorporating MIPs for illicit drug sensing/detection.

### 4.2. MIPs for Sensing/Detection of Illicit Drugs by Optical Techniques

Despite chromatography-related combined methods, MIPs have been incorporated into a wide range of optical devices for illicit drugs detection, such as **Raman** spectroscopy [146], **nuclear magnetic resonance [147]**, and total reflectance **Fourier** transform-infrared (ATR-FTIR) or near-infrared spectroscopy (NIR) [148,149,150]. As a fast, non-destructive technique for in-situ detection of various hazardous-type compounds, Raman spectroscopy/scattering (RS) is used for cannabinoid quantification. One of the leading causes of cannabis intoxication is the presence of recreational Trans-Δ^9^-tetrahydrocannabinol (THC), used very often by young people; due to its legalization, the task to detect THC at trace levels concentrations in complex gas matrices containing other water vapors is extremely challenging. Yeganegi and Hoor far [151] successfully detected THC by loading MIP nanoparticles prepared by precipitation polymerization and RS technology as the spectroscopic transducer. This Raman technique allowed the label-free monitoring of THC based on a single identifying Raman peak (a peak at 1614 cm^−1^ in the MIP spectrum), leading to a limit of detection of 250 ppm. The sensor’s selectivity was determined by exposing the MIP sensor to different analytes, including Cannabidiol CBD, ethanol, and acetone. To detect THC in indoor air quality assessment or monitoring emissions in cannabis cultivation facilities, the same group [152] developed a highly selective microfluidic (microchannels and MOS detectors) integrated metal oxide gas sensor using the nanoMIPs. The sensitivity and selectivity of coated microfluidic integrated gas sensors were evaluated by exposure to THC, CBD, methanol, and ethanol analytes in 300–700 ppm at 300 °C, with 96.3% accuracy channel response data. 

These techniques are helpful but inherently semi-quantitative; thus, this review section summarizes several **colorimetric and fluorescence** sensors integrating MIPs, which have shown excellent potential in the recognition and detection of various illicit drugs, specifically methamphetamine; to date, only a few publications have been reported for illicit drugs MIPs-specific colorimetric and fluorescence detection (Table 5). Colorimetric sensors have emerged as favored tools to detect illicit drugs and opioids [145] based on their attributes, such as low cost, easy preparation, and no need for skilled specialists across various applications, ranging from pharmaceutical and point-of-care (POC) diagnostic to forensic settings. Recently, POC devices utilized for on-site drug detection in a variety of clinical conditions for reliable, easy, and fast health monitoring can be integrated with colorimetric methods. Thus, the group of Akhoundian and Alizadeh developed POC non-enzymatic and portable MIP-based colorimetric sensors for ephedrine [153] and methamphetamine [154] in urine samples. Digital image colorimetry (DIC) as a combination of colorimetric reaction product and digital photo analysis was applied in both cases, considering the evaluation of the RGB (red, blue, green) color intensities.

The incorporation of a MIP into a colorimetric tool has many approaches, with various mechanisms being employed to achieve the desired colorimetric detection and dramatically enhance the sensitivity and selectivity, including MIP nanoparticles dye displacement assays [155,156], quantum dots (QDs) [157,158,159], or others (mimetic enzymes, polymeric micelles, ligand-receptor binding). In essence, SDC, as a method of converting MIPs into a visual analytical tool by prebinding a dye molecule (such as malachite green) into the nanocavities available in the MIP, was used by some authors [155]. One of the fewest works on opioid detection in biological samples by MIP optical sensors is a POC fiber optic sensor fabricated by Liu and coworkers [156]; this long-period grating (LPG) sensor functionalized with nanoMIPs allows the detection of fentanyl (due to identical binding sites to carboxyl-fentanyl), and carboxyl-fentanyl, the major metabolite of butyrylfentanyl presented in blood or human plasma. These sensors are popular label-free detection techniques that detect biomolecular interaction via a change in surface refractive index (RI). The sensor they developed exhibited a gradual response over increasing concentrations of carboxyl-fentanyl from 0 to 1000 ng/mL, with LOD down to 50 ng/mL and high selectivity upon morphine and cocaine. The use of QDs as fluorescent nanomaterials (of <10 nm in size) is well known for designing optical sensors for highly sensitive measurements of hazardous compounds. Among them, graphene quantum dots (GQDs), possessing good water dispersibility, tunable fluorescence properties, biocompatibility, and low toxicity and photo-stability, have shown excellent potential for replacing traditional QDs in detecting methamphetamine, as reported by Masteri-Farahani et al. [159]. Fluorescence-based detection methods as photoluminescent nanosensors have received considerable attention thanks to their remarkable sensitivity and versatility in terms of transduction schemes.

**Table 5:** MIPs for colorimetric and fluorescence sensing of illicit drugs and their characteristics.

Sensor Type/Detection Method	MIP Polymerization Method	Sensor Modification	Target	LoD ^1^ (M)	Linear Range	Real Samples	Refs.
Colorimetric/Non-enzymatic	Precipitation	MIP and CS2-Cu(II) complex	Ephedrine	0.6 μM	1–100 μM	Urine	[153]
Colorimetric/Non-enzymatic	Precipitation	MIP and ninhydrin	Methamphetamine	1.44 μM	5–100 μM	Urine	[154]
Colorimetric/UV Spectroscopy	Bulk	MIP-Based Dye DC	Amphetamine	57 μM	0.01–0.20 mg mL^−1^	Urine	[155]
Fibre Optic- long period grating (LPG)	SPE	nanoMIPs/PG array via EDC/ NHS coupling	Carboxyl-fentanyl	0.13μM	0–1000 ng mL^−1^	Blood, human serum	[155]
Fluorescence	Free radical	AuZnFeSeSQDs@MIP core/shell nanocomposite	Levamisole	0.05 μM	0.5–100 μM	Mixed drug containing cocaine	[157]
Fluorescence	Free radical	AuZnCeSeS QDs-MIP nanocomposite	Methamphetamine	0.02 nM	0.05–50.000 nM	Urine	[158]
Photoluminescent/Fluorescence	photoluminescence	GQDs-MIP	Methamphetamine	12 nM	5–50 μM	NA ^2^	[159]

^1^ When appropriated, the LOD (*w*/*v*) was recalculated in molarity (MW carfentanil = 394.512 g mol^−1^); ^2^ NA not available.

### 4.3. MIPs for Sensing/Detection of Illicit Drugs by Electrochemical Methods

This review section highlights the most recent innovations in electrochemical sensors for illicit drugs, emphasizing opioids within bioanalytical, forensic, and environmental applications, and describes other electrochemical sensing strategies previously reported. Table 6 gives a summarized overview of figures of merits, including the electrode type, electrode modification, electrochemical method, target molecule, linear range, limit of detection (LOD), and recoveries of the electrochemical sensors studied. 

Electrochemical sensors have become crucial analytical techniques for illicit drugs simultaneous analyses with desirable advantages, including high sensitivity and specificity, user-friendly interfaces and straightforward readouts, portable and short response time, mostly when modified with MIPs and MOFs [160,161,162] or multi-wall carbon nanotubes (MWCNTs) [163,164,165] graphene and graphene oxide (GO) nanomaterials possessing high stability, specific surface area, and conductivity [162,166,167,168], for sensing drugs from Schedule I and II (CBD, THC, MDMA, cocaine, amphetamine, fentanyl). It is relevant to note that all the strategies reported for drug sensing rely on cross-linked and electroactive polymers that are often developed using electropolymerization methods. Recently, MOFs are a group of tridimensional porous crystalline linked via organic ligands and metal ions, with highly ordered polyporous structure and multifunction properties; among many zirconium MOF, UiO-66 is made by hexameric Zr6O32 units and 2-amino-terephthalate linkers exhibiting good performances in many fields like adsorption, capacitor, catalysis, medical, and environmental detection [162].

MIP films on different electrodes (Pt, Au-E, GPE) were synthesized via electropolymerization with Amino acids/indazole-based cannabinoids, 3,4-methylene dioxyphenethylamine (MDPEA) and oxycodone as the template molecules, respectively, which can be used to quantify SCs in street preparations [169], ecstasy in biological samples [170] and oxycodone in wastewaters [171]. Moreover, nanoMIPs were used to sensitively (up to nanomolar) detect cocaine [172] and fentanyl compounds [173,174]. Magnetic MIP nanoparticles (MMIPs) have attracted a great deal of interest due to their great potential application especially in biosensors, minimizing matrix effects in complex samples. Fe_3_O_4_ nanoparticles are the most commonly used magnetic material due to easy fabrication, low toxicity, and, most importantly, allowing convenient modification with abundant hydroxyls on the surface. MMIPs can be easily synthesized by core-shell technique (silica-coated magnetite nanoparticles Fe_3_O_4_@SiO_2_) and coprecipitation polymerization [156,173].

Lately, capacitive sensors have become a popular tool for the detection of illicit drugs, primarily for environmental applications [175,176,177], due to the lack of sample pretreatment or large sample volumes yet of high-throughput screening via fast response time and real-time data processing. Multiplex capacitive detection systems for illicit drugs were rarely developed using natural and artificial recognition elements on the transducers, mostly gold electrodes [177].

**Table 6:** Overview of the MIP-based electrochemical sensors for illicit drug detection and their analytical performances.

Electrode Type/Detection Method	MIP Polymerization Method	Electrode Modification	Target	LoD ^1^ (M)	Linear Range	Real Samples	Recovery Rate (%)	Refs.
GCE/CV, DPV	Electro polymerization	Zn/La^3+^/MOF/MIP	Buprenorphine BUP	0.0021 μM	0.0079–0.0992 μM	Blood	99.1–100.2	[161]
SPCE/CV, HPLC	Precipitation	graphene-UiO-66 composites/MMIP	Cannabidiol CBD	0.05 μM	5–100 μM	CBD product	99.5–99.8	[162]
SPCE/DPV	Electro-polymerization	MIP/MWCNTs	THC	0.54 nM	0–3150 nM	Human blood plasma	99.75	[163]
SPEISE/Potentiometric	Precipitation	MIP/MWCNTs	Pholcodine PHO	0.25 µM	5.5 µM–0.01 M	Serum	91–95.5	[164]
ITO/DPV	Sol–gel and electropolymerization	pyrrole@sol-gelMIP/fMWCNT	Naloxone	0.02 µM	0–12 µM	Urine	>88	[165]
SPCE/UPLC-MS/MS	SPE/UV radiation	Chitosan/RGO/Electroactive nanoMIPs	MDMA	1.6 nM	1–200 nM	Street probes	92–99	[166]
SPPE/DPV	SPE/UPLC-MS	nanoMIPs/graphene by 3D printing andnanoMIP/silane by drop-casting	Amphetamine	68 and 37.6 nM	75–220 nM and 25–220 nM	Human plasma and street	NA ^2^	[167]
GCE/SWV	Electropolymerization	MIM-ErGO	Fentanyl	1.28 nM	0.0038–1.72 μM	Human serum	97.0–110	[168]
Pt/DPV	Electropolymerization	polyacrylate-based MIP	Aminoacids/indazole-based cannabinoids	0.01 mM	Up to 0.8 mM	Simulated pills and smoking mixtures	70–115	[169]
Au-E/DPV	Electro-polymerization	polydopamine-based MIP	Homopiperonylamine (MDPEA)	54 nM	0.1–7.5 μM	Urine	99.27–108	[170]
GPE/SWV	Electro-polymerization	o-phenylenediamine-based MIP	Oxycodone	1.8 ± 0.239 nM	0.4–5.0 nM	Wastewater	96.0–102.5	[171]
Au/EIS	SPE	nanoMIPs	Cocaine	0.70 nM	0.30–147 nM	Diluted cocaine	NA ^2^	[172]
CPE/SWV	Precipitation	Magnetic Fe_3_O_4_ nanoMIPs	Sufentanil	0.02 μM	0.001–0.06 μM	Urine and plasma	96.0–102.5	[173]
SPPE/DPV	SPE/UV radiation	nanoMIPs	Fentanyl	0.28 nM	5–60 nM	Human plasma	NA ^2^	[174]
Au transducers/Capacitance	Photoinitiatedemulsion	MIP/flow-injection	4-methyl-5-phenylpyrimidine (4M5PP)	80 μM	100–3000 μM	Wastewater	95–101	[175]
Au electrodes/Capacitance	Bulk polymerization	immobilized MIPs	benzyl methyl ketone (BMK)	1 μM	50 to 1000 μM	Spiked tap water and real water	NA ^2^	[176]
Au/Multiplex capacitive, CV, and optical microscopy	Electropolymerization	MIP, Poly-tyramine/	Amphetamine	50 μM	NA^2^	Sewage and tap water	NA ^2^	[177]

^1^ When appropriated, the LOD (*w*/*v*) was recalculated in molarity (MW MDMA = 193 g mol^−1^; MW THC = 314.45 g mol^−1^; MW cocaine = 339.816 g mol^−1^); ^2^ NA not available.

## 5. MIPs Designed for Biological Agents Assessment

Biological agents include infectious agents such as bacteria, viruses, fungi, and insects [178]. Despite advancements in understanding and treatments, biothreat agents continue to pose a considerable public health risk. Early detection is essential for effective prevention and protection measures. The primary challenge in developing detection systems lies in enhancing the sensitivity of instruments or methodologies to identify the concentration levels at which microorganisms can induce disease in humans. Additionally, it is essential to verify the presence of these pathogens across diverse matrices. Furthermore, the detection system must be designed to be portable, user-friendly, and capable of detecting multiple agents simultaneously.

Table 7 below illustrates a selection of current and emerging methods for detecting biological agents [179].

Humanity has recently learned some important lessons from the COVID-19 pandemic. Even as the impact of the pandemic appears to be subsiding, it is essential to recognize that reducing the global burden of bacterial infection-related mortality remains a significant and pressing public health priority for humanity. While not classified as biological weapons (like *Bacillus anthracis*), it is worth noting that five pathogens, namely *Staphylococcus aureus* (*S. aureus*, a gram-positive bacterium that colonizes the nasal passage and skin, causing invasive disorders such as bacteremia, endocarditis, osteomyelitis, pneumonia, and skin infections; commonly treated with antibiotics like oxacillin and nafcillin [207]), *Escherichia coli* (*E. coli*, gram-negative bacterium including serotypes like *E. coli* O157:H7, which can cause severe intestinal infections; treatment includes ciprofloxacin and tetracycline [208]), *Streptococcus pneumonia* (*S. pneumonia*, encapsulated Gram-positive bacterium causing pneumococcal diseases such as pneumonia, meningitis, and sepsis, particularly in children; treated with penicillin and vaccines [209]), *Klebsiella pneumoniae* (*K. pneumoniae*, encapsulated Gram-negative bacterium implicated in pneumonia, urinary tract infections, and liver abscesses, and treated with broad-spectrum antibiotics like carbapenems [210]), and *Pseudomonas aeruginosa* (*Ps. aeruginosa*, a motile Gram-negative bacterium causing various infections including those of the skin, eyes, ears, respiratory and urinary tracts, and gut-derived sepsis; treatment includes cefepime or gentamicin [211]), accounted for 54.9% of deaths associated with bacterial pathogens in 2019 [212].

The capability to differentiate between dangerous and non-dangerous biological agents is necessary for various military and civilian purposes. The deliberate release of viruses, bacteria, toxins, or other harmful agents with the intention of causing illness or death in humans, animals, or plants is known as a bioterrorism attack. Many countries have used biological weapons in the past, leading to bioterrorism becoming a significant issue since the 1980s. While these agents occur naturally, they can be modified to enhance their capacity to cause harm. Biological agents can be disseminated through the air, water supplies, cities, airports, or food. 

The most notorious biological agents include anthrax, smallpox, botulinum toxin, bubonic plague, viral encephalitis, food threats such as *Salmonella* species, *E. coli* O157:H7, *S. aureus*, *Staphylococcal enterotoxin B*, *typhus* (*Rickettsia prowazekii*), water supply threats like *Cryptosporidium parvum*, and others [179]. Notably, some bioterrorism agents, such as the smallpox virus, can spread from person to person, while others, such as anthrax, cannot. Perpetrators may opt for biological agents due to the challenge of detecting them and their potential to cause delayed illness, ranging from hours to days.

Healthcare professionals and the public health system must be prepared to manage a range of biological agents, including rare infections. High-priority agents are organisms that pose a significant threat to national security due to their high mortality rates and potential impact on public health. These agents are easily transmissible between individuals, have the potential to cause societal tensions and public panic and require special measures to prepare the public for health emergencies. 

It is important to note that the development, production, procurement, transfer, stockpiling, and use of biological agents and toxins as weapons are strictly prohibited by the Biological Weapons Convention (BWC). This convention serves as a pivotal instrument in the international community’s comprehensive strategy to prevent the spread of weapons of mass destruction (WMD). Through its stringent provisions, the BWC has successfully fostered a compelling international standard that unequivocally denounces the possession and use of biological weapons. This robust framework has been instrumental in promoting global security and stability by mitigating the threats associated with biological warfare and encouraging adherence to established international protocols. Understanding and categorizing biological warfare agents (BWAs) is crucial for national security and global cooperation. 

The U.S. Centers for Disease Control and Prevention (CDC) has classified BWAs into three categories A (the highest priority threats, the most concerning, including pathogens like anthrax and smallpox), B (agents which demand attention due to their moderate ease of dissemination, including diseases like brucellosis and tularemia), and C (the emerging threats, often underestimated but have the potential to cause severe impacts, e.g., Nipah virus and hantaviruses). 

Table 8 also provides a description of the most prevalent biological warfare agents, along with their main attributes, symptomatology, and recommended treatments.

The escalating security concerns and the ongoing battle against terrorism underscore the critical need for advanced biosensors capable of rapidly detecting bio-warfare agents (BWAs) to safeguard military and civilian defense interests. It is widely recognized that while legislation is a crucial component, it alone cannot fully ensure security and homeland defense. The identification and neutralization of BWAs play a pivotal role in safeguarding against potential threats. Therefore, it is imperative to implement comprehensive strategies for early detection and rapid response to such biosecurity risks.

### 5.1. MIP Versatile Tools for Detecting Biological Agents

The following section of the review article provides an overview of advancements in the development of cost-effective, durable, portable, and highly selective biosensing MIP-based systems for the efficient detection of various pathogens. The analytes of interest encompass a diverse array of microorganisms, pathogens, toxins, as well as specific biological warfare agents or biomarkers.

Another essential aspect discussed herein refers to innovative technological approaches that facilitate the development of devices aimed at mitigating bioterrorism risks and reducing the threat of biological agents. The focus will be on the application of molecularly imprinted polymers in conjunction with various detection techniques, including but not limited to electrochemical impedance, electrochemiluminescence, photoluminescence, and additional optical or physical sensing methods.

Optical sensors operate by assessing variations in the optical characteristics of materials. The interaction between the target analyte and the recognition molecule induces a measurable optical change, which is subsequently transformed into an electronic signal [226]. Numerous efforts have been made to identify microbial contaminants through optical sensing techniques, including colorimetric methods [227], fluorescence detection [228], interferometric approaches [229], SPR [230], Raman spectroscopy [231], and SERS [232]. Alternatively, electrochemical sensing platforms have garnered significant interest in the identification of microbial contaminants. These platforms are characterized by their relative simplicity, ease of design, low LOD, rapid response times, high sensitivity, and improved selectivity [226]. Prevalent electrochemical techniques employed for the detection of pathogens include voltammetry, amperometry/potentiometry, and impedance spectroscopy [233].

**Figure 6:**
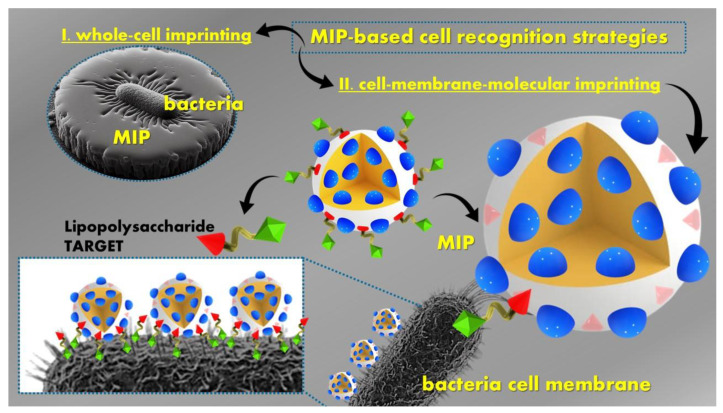
MIP-based cell recognition strategies.

The selection of imprinting strategies for the preparation of MIPs depends on the dimensions of the template used. In the case of bulk imprinting, recognition sites are distributed throughout the polymer matrix, facilitating the uptake and release of smaller molecules. However, this method is sometimes inadequate for bacterial imprinting due to the larger size of the analytes, which may remain in the densely cross-linked matrix [234]. Consequently, surface imprinting emerges as a more suitable technique for the imprinting of bacterial targets. To identify a cell, MIPs must be engineered to exhibit a particular binding affinity for distinct molecules present in the cell membrane, such as proteins, lipids, and glycans, or for the collective characteristics of the cell membrane itself. Thus, the contemporary approaches for MIP-based cell recognition (Figure 6) can be broadly classified into two main categories: whole-cell imprinting and cell-membrane-molecular imprinting strategies [234,235,236].

The whole-cell imprinting technique offers a seemingly simple method, yet it presents considerable challenges. These challenges stem from several factors, including the complex structures, substantial sizes, delicacy, and fluidity of cells that require meticulous attention. For microorganisms like bacteria and viruses, direct imprinting of entire cells is generally achievable. However, due to the significant size and complexity of cells relative to individual molecules, surface imprinting becomes crucial for effective cell identification. The cell-membrane molecular imprinting method closely parallels traditional molecular imprinting, which targets molecules as the primary entities. Therefore, the careful selection of appropriate cell membrane molecules for the imprinting process is essential to ensure successful cell recognition [235].

The following paragraphs describe a selection of the latest notable achievements in the detection of pathogenic agents, including BWAs, utilizing MIP-based sensors. Additionally, Table 9 offers a concise overview of significant research on MIP-based sensing techniques for biological agents, facilitating a straightforward comparison of their effectiveness.

Emphasizing the importance of defensive biological research is essential for enhancing prevention and preparedness strategies against bioterrorism. In the field of sensor technology, optical sensors have emerged as a focal point, particularly across various subcategories defined by their detection mechanisms. Extensive investigations have been conducted on a diverse array of luminescent materials for the advancement of biosensors. Among the notable threats posed by spore-forming bacteria, *B. anthracis* stands out due to its potential as a bioweapon, resulting in severe consequences for both human and animal populations. This bacterium, along with other species within the Firmicutes phylum, can be detected using specific biomarkers, notably dipicolinic acid (DPA), which is characteristic of *B. anthracis* [237]. Solmaz Norouzi et al. [237] described a red-emissive carbon nanostructure anchoring a molecularly imprinted Er-BTC (1,3,5-benzene tricarboxylic acid)—MOF as a fluorescence biosensor for the visual determination of DPA.DPA is recognized as a significant ligand for lanthanoids [237]. The unique combination of high affinity, intrinsic luminescent characteristics, photostability, and adjustable pore sizes positions Erbium MOFs as optimal materials for the detection of the DPA biomarker. The integration of CDs with a high luminescence quantum yield (QY) as guest fluorescent nanoparticles within Er-MOFs, achieved through a straightforward and effective methodology, could pave the way for innovative developments. The synergy of this substrate with MIP biosensors based on MOFs presents a compelling strategy for creating sensitive and cost-efficient systems for selective fluorometric detection of DPA. The proposed fluorometric visual biosensor [237] exhibits extensive linear detection ranges for DPA (10–125 μM), with LOQ and LOD of 4.32 and 1.28 μM, respectively. Successful monitoring of DPA has been demonstrated in real samples, including tap water and urine. This integrated selective paper-based nano-biosensor, in conjunction with smartphone signal recording capabilities, shows significant potential for advanced practical applications, encompassing fluorometric and colorimetric detection in healthcare, environmental monitoring, food safety assessments, and point-of-care testing [237]. The results obtained by Solmaz Norouzi et al. [237] indicated that the recoveries achieved were satisfactory, ranging from 95.0% to 104.46%. Additionally, the relative standard deviation (RSD) was maintained at 3.96%, which supports the reliability and prospective use of the developed methodology in monitoring DPA as a biomarker for anthrax.

**Table 9:** Overview of MIPs for biological agents sensing applications.

Sensor Type	Polymerization Method	Substrate/Sensitive Material	Target	Detection	Performances	Refs.
Optical	Free radical polymerization by thermal initiation	*E. coli*—stamp imprinted Poly(styrene-co-divinylbenzene)	*E. coli* and *B. cereus*	confocal Raman Microscopy and AFM	- Confocal Raman Microscopy was utilized to differentiate and visualize bacteria, imprints, and polymers; - classification accuracy of 95% for the Raman spectra	[238]
Electrochemical	Electro-copolymerization of aniline and p-aminophenyl boronic acid	Binding sites of boronic acid group	*E. coli* K-12	MIP film reversibly binds glycan on *E.coli* cell surface	- *E.coli* was determined by MIP film-coated electrode up to 2.9 × 10^4^ cells-mL^−1^; - sensitivity 0.46 [nF/log(cells-mL^−1^)]	[239]
Electrochemical sandwich sensor	One-step electro-copolymerization of 3-thiopheneethanol (TE) monomer and *S. aureus* template	Sandwich-type electrode: Gold nanoparticles modified with aptamers and electroactive 6- (Ferrocenyl)hexanethiol (Fc) /bacteria-imprinted polymer film/Glass carbon electrode	*S. aureus*	Dual recognition by the bacteria-imprinted polymer film (BIF) and Aptamer	- quantitative detection of *S. aureus*; - good selectivity; - ultrasensitive detection of *S. aureus* down to a single cell in PBS; - LOD in milk samples 10 CFU-mL^−1^ of *S. aureus*	[240]
Optical/ Surface imprinting	Free radical polymerization method by thermal initiation	Carboxylic-terminated polystyrene (CPS) microparticles/monomers: acrylamide, methacrylic acid, methyl methacrylate, N-vinylpyrrolidone Dihydroxyethylenebisacrylamide	*E. coli* OP50	Selective entrapment of *E.coli* OP50	- uptake ratio of bacteria (10^4^ cells-mL^−1^); - the optimum uptake ratio of *E. coli* was approximately 74%, which was achieved using 10^3^ MIP-MPs-mL^−1^	[241]
Electrochemical EIS-	One-step electro-polymerization	Conductive poly(3-thiopheneacetic acid) deposited on gold electrode	*S. aureus*, *L. monocytogenes*, *E. coli* and *S. paratyphi*	Selective detection of *S. aureus* from artificially contaminated milk	- dynamic response within 10 min; - high selectivity for *S. aureus*; - low LOD of 2 CFU- mL^−1^; - wide linear range from 10 to 10^8^ CFU- mL^−1^	[242]
Electrochemical EIS-	Electro-copolymerization of the template and TE monomer on a GCE	Bacteria-imprinted polythiophene (3-thiopheneethanol—based film)	*S. aureus*	Identifying *S. aureus* from multi-bacterial strain mixtures.	- linear range of 10 to 10^7^ CFU-mL^−1^; - low detection limit of 4 CFU-mL^−1^; - excellent selectivity for *S. aureus*; - applicability in the analysis of real lettuce and shrimp samples	[243]
Electrochemical EIS-	Electro-polymerization	Electrochemically fabricated poly(3-aminophenyl boronic acid)—based MIP deposited on gold disk electrodes	*S. epidermidis*	Label-free detection -	- linear response over the range of 10^3^–10^7^ CFU-mL^−1^; - high selectivity for *S. epidermidis;* - reversibility of the cis-diol-boronic group complex	[244]
Electrochemical EIS-	Electro-polymerization	Poly(o-phenylenediamine) on glass carbon electrode	*E. coli* O157:H7 and *S. aureus*	Dual bacteria-imprinted polymer (DBIP) -	- low detection limit of approximately 9 CFU mL^−1^; - effectiveness in detecting the target bacteria in apple juice; - recovery rates for *E. coli* and *S. aureus* ranged from 86.86% to 98.40% and 81.36% to 100.58%	[245]
Electrochemical EIS-	One-step electro-polymerization	Bacteria-imprinted polypyrrole (BIP) film on -GCE- surface	*E. coli* O157:H7	Noncavity imprinted sites situated at the surface of the PPy matrix	- LoD for *E.coli*- 10^3^ CFU- mL^−1^; - high selectivity; - recoveries 96.0–107.9% (RSDs < 4% for *E. coli* in real samples (drinking water, apple juice, and milk)	[246]
Electro-chemi-luminescence (ECL)	Electro-polymerization	Polydopamine (PDA) surface imprinted polymer (SIP) film - and nitrogen-doped graphene quantum dots	*E. coli* O157:H7	*E.coli* detection and quantitative measurements	- 10^1^–10^7^ CFU-mL^−1^ *E. coli* in water; - *E. coli* can be detected via the ECL signal of N-GQDs with K_2_S_2_O_8_ as the co-reactant	[247]
Fluorescence-colorimetric dual-mode	Ionic polymerization	Fe_3_O_4_ coated with carbon quantum dots; Phenolphthalein was coated with ZIF-8 and then surface-modified with EV71 aptamers to specifically bind to the target	*virus* *EV71*	Aptamers introduced into the imprinting layer to enhance the recognition of the target virus	- LOD fluorescence 8.33 fmol-L^−1^; LOD visualization 2.08 pmol-L^−1^	[248]

Another study published in 2024, accomplished by M. Zolfaghari et al. [249], also focused on the detection of DPA as a biomarker in bacterial spores of *Bacillus anthracis*, but via a sensitive electrochemical MIP sensor. The electrochemical sensor developed by M. Zolfaghari et al. [249] consists of a simplistic design based on a glassy carbon electrode (GCE) that is modified with gold nanoparticles and an MIP. This configuration provides sensitive and selective binding sites for the detection of the DPA molecule. To fabricate this sensor, a suspension containing AuNPs was applied to the surface of the GCE using a micropipette, followed by the evaporation of water at ambient temperature to achieve the modified electrode. For MIP synthesis, for the polymerization process, which occurred in the presence of DPA, methacrylic acid (MAA) and ethylene glycol dimethacrylate (EGDMA) were utilized as the functional monomer and cross-linker, respectively, with 2,2′-azobisisobutyronitrile (AIBN) serving as the initiator. The MIP was synthesized through a bulk polymerization technique. Subsequently, the modified GCE was immersed in a suspension of both MIPs and non-imprinted polymers (NIPs), allowing the mixture to rest at room temperature for 30 min to facilitate the evaporation of ethanol from the GCE surface. The amperometric response exhibited a linear relationship with varying concentrations of DPA, specifically within 10^−14^ to 10^−9^ M and 10^−8^ to 10^−1^ M (R^2^~0.9956), with a LOD estimated at approximately 1.58 × 10^−8^ M [249].

Ribosome-inactivating proteins (RIPs) represent a class of compounds found in various organisms, including bacteria, plants, algae, and fungi. These proteins can be classified into three distinct types: type I, type II, and type III. Among these, type II is particularly significant, consisting of a chain that exhibits lethal rRNA N-glycosidase activity (the A-chain) and a lectin chain (the B-chain) that functions as a binding agent at the cell membrane receptor. Several highly toxic proteins derived from plants belong to the type II RIPs family, such as *Viscumin, Abrin*, and *Ricin* [250]. In this context, a molecularly imprinted polymer (MIP) designed for the biological warfare agent ricin was developed by S. Pradhan et al. [251] utilizing silanes (3-aminopropyltriethoxysilane as monomer and tetraethoxysilane as cross-linker). Ricin, a highly toxic substance derived from the seeds of the castor bean plant *Ricinus communis*, is recognized as a biological warfare agent (Table 8). The ricin molecule consists of two glycoprotein chains, designated as A and B, which are of comparable size (molecular weight approximately 62 kDa) and are linked together by a disulfide bond [251,252]. Extraction of the protein template was a straightforward process, and the three-dimensional structure of the protein was preserved within the silane-based MIPs, yielding an imprinting efficiency of 1.76 [251]. The recognition of ricin by the Ricin-MIP was achieved through the adsorption of ricin from an aqueous solution. Additionally, the Ricin-MIP exhibited a 10% interference from the structurally analogous protein, abrin [251].

S. Nasirahmadi et al. [250] developed molecularly imprinted polymers (MIPs) for the identification of viscumin, a plant-derived protein toxin found in mistletoe. In this study, molecular imprinting was employed to generate selective recognition sites for the 9-mer peptide epitope of viscumin (ML1 protein) in standard media. The MIP was obtained using UV in-situ polymerization via micro-contact techniques. The most stable complex was achieved with a template-to-functional monomer molar ratio of 1:4. The MIP exhibited high affinity for the epitope, along with favorable limits of detection (LOD) and quantification (LOQ). Specifically, the designed nano-biosensor demonstrated a LOD of 0.117 ng/mL and a LOQ of 0.517 ng/mL in PBS buffer. These detection levels were 0.5 ng/mL and 0.8 ng/mL in urine and 1.25 ng/mL and 5 ng/mL in human blood fresh frozen plasma, where ricin served as the closest homolog to viscumin (ML1) at a fixed concentration ratio of 12:1. The optimal detection time was 8.0 min, and the preferred pH was 7.4. Overall, this MIP-based nano-biosensor holds promise for applications in diverse, complex environments due to its efficiency.

The review published by Yongbiao Hua et al. [253] provides a comprehensive overview of the recent developments in the utilization of MIPs for the detection of various mycotoxins (e.g., aflatoxins, patulin). The authors detail the construction of diverse MIP-based sensors, which can be categorized into optical sensors (such as fluorescence, phosphorescence, SPR, and SERS), electrochemical sensors (including both electrochemical and photoelectrochemical types), and piezoelectric sensors aimed at identifying mycotoxins in a range of food samples. The efficacy of these MIP-based sensors in detecting mycotoxins is notably enhanced through the integration of additional materials, such as noble metals (like platinum and gold) and carbon-based substances (including CNTs, MWNTs, and QD). The development of MIP composites with these materials has emerged as a promising strategy due to their numerous advantages, which encompass improved electronic and conductive properties, increased catalytic activity, and simplified analyte detection. Among the various sensor types, MIP-based electrochemical sensors demonstrate superior sensitivity, particularly in terms of limits of detection for most mycotoxins [253]. Furthermore, the combination of MIP-based sensors with advanced technologies (e.g., handheld devices, microfluidic systems, smartphone applications, and lab-on-a-chip platforms) holds significant potential for providing reliable on-site analysis of mycotoxins in complex food matrices and real-world scenarios.

### 5.2. Integrating Bacterial Sensing and Intrinsic/Stimulus-Driven Decontamination in MIPs

**Figure 7:**
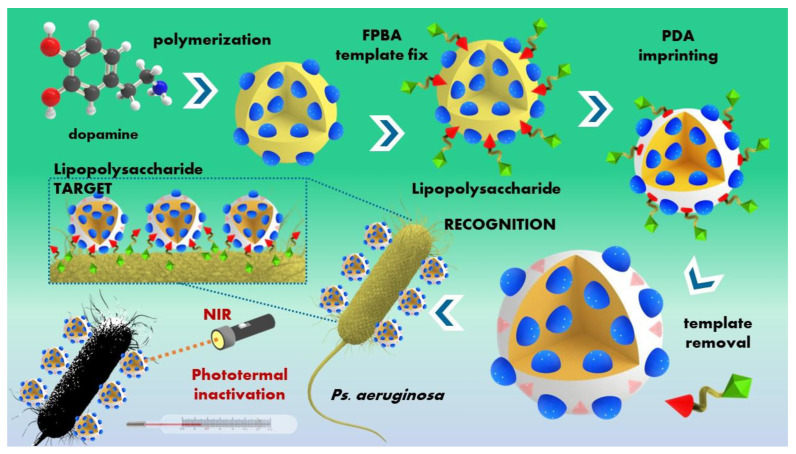
Graphic illustration of MIP preparation, *Ps. aeruginosa* recognition, and photothermal inactivation—adapted from [254].

Bacteria-targeting materials with a complementary photothermal effect are a significant area of interest in biotechnology. One particularly promising material for this purpose is molecularly imprinted polymers (MIPs). These polymers have been thoughtfully designed to identify and selectively bind to target bacteria. When endowed with a photothermal effect, they have the potential to assist in the selective destruction of the bacteria. In this context, Zhang et al. [254] developed lipopolysaccharide (LPS) imprinted photothermal molecularly imprinted polymers (PMIP) for the recognition and elimination of *Ps. Aeruginosa* (Figure 7). The MIPs reported in this study demonstrated significant optical absorption in the 808 nm region of the light spectrum, which can be attributed to the absorbance of dopachrome and dopaindole oxidized from dopamine. Upon exposure to an 808 nm laser (2 W cm^−2^) for 10 min, the temperature of PMIP rose to 42.9 °C, 52.7 °C, and 58.1 °C for particle concentrations of 100 μg mL^−1^, 200 μg mL^−1^, and 300 μg mL^−1^, respectively. The observed increase can be attributed to the photothermal effect induced by the PMIPs combined with their LPS binding abilities. Consequently, in the presence of PMIP and NIR irradiation, only a minimal number of *Ps. aeruginosa* colonies were able to survive [254]. 

Shao et al. [255] also described optimized LPS-MIPs and demonstrated their abilities for selective recognition at trace levels of the target Gram-negative bacteria from whole blood samples. Further, the imprinted polymer nanoparticles were encapsulated with gold nanorods (gold nanorods were incorporated during the polymerization process) for selective microbial inactivation of bacteria via photothermal treatment, reporting a significant temperature increase from 27.6 to 49.3 °C at a concentration of 3 mg/mL with a light irradiance of 2 W/cm^2^. Under light irradiation, the imprinted polymer nanoparticles encapsulated with gold nanorods were able to selectively and efficiently kill *E. coli* from a blend of three different types of strains comprised of *E. coli*, *Yeast*, and *Bacillus subtilis* by targeting the Gram-negative bacteria through LPS-imprinted cavities. Thus, in this case, the incorporation of gold nanorods into imprinted nanoparticles allowed for selective bacteria inactivation based on the photothermal effect [255]. 

Yang et al. [256] developed a novel bacterial imprinting technique using interfacial biomimetic mineralization. Their approach successfully targeted antibiotic-resistant bacteria (ARB) without harming beneficial bacteria in wastewater. By integrating whole-cell imprinting and epitope imprinting strategies, they synthesized magnetic bacterial imprinted polymers (BIPs) specifically for ARB. The BIPs precipitated on the surface of antibiotic-resistant E. coli cells demonstrated good photothermal stability (31.7% conversion efficiency) and potential for selective anti-pathogen applications in water purification, biological treatment, and environmental bioremediation.

To summarize, in addition to their sensing abilities, MIP-based materials with enhanced target selectivity and photothermal effectiveness may prove to be beneficial in mitigating bacterial infections and enhancing the management of illnesses resulting from pathogenic microorganisms.

## 6. Conclusions, Emerging Trends, and Future Perspectives

This review aids the readers in grasping the increasing trend for sensor development, which represents a response to the necessity for more efficient tools in detecting hazardous compounds and for taking concrete actions to decontaminate the air, water, and land before becoming a much more costly problem of biosafety and biosecurity. The recent spike in literature reports describing new sensing tools and methods is also associated with the increase in the number and types of hazardous synthetic compounds.

In this context, up to date, various kinds of optical and electrochemical sensors that allow miniaturization, portability, and scalability were developed to detect chemical and biochemical agents, illicit drugs, or explosives. Yet, to solve other stringent issues of sensors, i.e., sensitivity and specificity towards target compounds, MI was approached, as referenced by this review. Although the concept of MIP is quite historical, it becomes meaningful when coupled with nanotechnology and analytical chemistry. Thus, analyzing the recent literature from 2020 to 2024, significant progress was made in detecting selectively the various types of hazardous structures by implementing MIP-based sensors. However, there is much space for improvement, especially since most studies follow the classical step-by-step optimization procedures. For instance, very few studies make use of available knowledge for experiment design or molecular modeling and statistical/quantum mechanics simulations that help construct a more rational experimental procedure to optimize the target parameters of interest. Therefore, understanding how MI can help improve the sensitivity and selectivity of sensors is a work in progress.

One of the toughest challenges an analytical chemist is faced with consists of handling very low concentrations of analytes in complex environments, particularly under the strong influence of humidity and interfering compounds. MIPs have garnered significant interest, but there are a series of conditions and difficulties when considering advancing to real-life applications [257,258]. Ghulam Mustafa et al. described several MIP Sensors that could be suitable for real-world applications [257]. Covering food safety [73], environmental monitoring, or public health fields [259], MIP sensors lab-based technologies can be successfully translated to commercialization. In this regard, Joseph W. Lowdon and co-workers [260] summarized the striking success stories of MIP applications via developing companies, from pioneering MIP Technologies and Supelco (now Merck) to MIP Diagnostics. The latter developed nanoMIPs, which were further integrated into gold screen-printed electrodes for the impedimetric detection of cocaine at trace levels. Other MIP sensor enterprises, such as PalmSens and EPI SISTEM, are focusing on cost-effective portable electrochemistry interfaces and small industrial solutions, respectively. Nonetheless, although implementation of these materials in the real world exists, commercializing the MIP devices is still critical and requires ongoing efforts [261].

Other future challenges for developing cost-effective and scalable MIP-based sensors are related to the most valuable feature of MIPs—specificity. Ideally, an MIP should have no or minimum cross-reactivity with other resembling species to reduce false-positive responses. Traditional methods of MIP production are often labor-intensive and time-consuming, which limits their application on an industrial scale. Future research should prioritize the development of more efficient and cost-effective synthesis methods. In other words, the more specific the MIPs are the less versatile to recognize structure-similar compounds. This would mean that for detecting with accuracy a single analyte, a specific/unique electrode is needed. In economic terms, this translates into higher production costs. For overcoming this economic challenge, dual/multi-molecular imprinting may be of help. According to some recent studies [262,263], dual-detection systems showed outstanding analytical performance for the simultaneous sensing of two different analytes. However, this approach is more adequate for templates with different structures, where the mutual interference between the analyzed species is minimal.

Artificial intelligence is also worthy of consideration for future studies, especially for predicting probable structures of drugs, explosives, and chemical agents that may emerge. Building sensing platforms with the use of virtual molecules [264] represents a future trend to counteract the increasingly high illegal transport of hazardous compounds.

Another hot spot for the use of MIPs targets viral decontamination. The proven capacity of MIP nanoparticles to act as synthetic receptors and to block the various functions of viruses, such as HIV and SARS-COV-2 [265,266,267], represent a valuable tool for onsite decontamination against biological threats. In the study of Xu et al. [265], MIP nanoparticles that can target and block the SWSNKS (3S), an epitope of the envelope glycoprotein 41 (gp41) of human immunodeficiency virus type 1 (HIV-1), to prevent subsequent cascade interactions directed toward the killing of CD4^+^T cells were proposed. At the same time, Batista and coworkers [266] developed silica core/shell MIPs as synthetic ACE2 receptors for blocking SARS-CoV-2 by using an epitope peptide comprising 17 residues (F486–G502) from the SARS-CoV-2 spike protein as a template molecule.

## Figures and Tables

**Table 2:** The most common CWAs and other threat agents, their attributes, and treatment.

CWAs		Main Properties/Toxicology	Treatment/Therapy	Refs.
**Blister or vesicants**	Sulfur mustard HD or Mustard gas SM, Nitrogen mustard HN1, lewisite L1, O-mustard	Damage occurs in the tissues; affects the lungs, eyes and produces skin blistering/ are relatively persistent	Anti-inflammatory agents (Dexamethasone), antioxidants, intracellular proteins like Dynasore, farnesoid receptor activation, Mesna for SM exposure, immunomodulators, British anti-Lewisite antidotes, and wound/tissue repair agents	[42,44]
**Nerve agents**	G-type: Sarin GB, Soman GD, Cyclosarin GF, Tabun GA; V-type O-ethyl S-[2-(diisopropylamino)ethyl] methylphosphonothioate VX, VG, VM, VE; A-type: A 230, A232, A234, A-242, A-262	Highly fatal due to their neurotoxicity; reacts irreversibly with cholinesterase; this results in acetylcholine accumulation, breakdown of the nervous system, convulsive status epilepticus, and death within minutes/ range in persistency	Antidotes like antimuscarinic agent atropine; benzodiazepines midazolam and ketamine; combined therapy for GD: allopregnanolone and ganaxolone; Pralidoxime; hydrophilic neurosteroids as anticonvulsants; parasympatholytic and neuroprotective agents for Novichok	[42,43,44,45]
**Incapacitating agents**	3-Quinuclidinyl benzilate BZ;	Prevents normal activity by producing mental or physiological effects	Antidotes like 7-MEOTA, atropine, and tacrine THA	[5,42,44]
**Irritants/riot control agents**	Tear aerosols CR, CS, CN, and OC pepper spray	Causes tearing and irritation of the skin, lungs, and eyes	TRPA1 with HC-030031 or A-967079 inhibitors for CS-induced skin injuries; Proliferating BPAECs; Bronchodilators like beta-2 agonists	[5,42,44]
**Choking agents**	Chlorine Cl, CG phosgene	Affects the respiratory tract and lungs	Limited or nonexistent therapeutic interventions (FXR activation on lung injury), oxidative stress, and fibrosis	[5,42,44]
**Blood or vomiting agents**	Cyanides DC and HCN, Adamsite, Arsine	Causes acute pain, nausea, and vomiting; prevents the transfer of oxygen to the body’s tissues	Hydroxocobalamin, cobalt (II/III) complex CoN4 [14]	[5,42,44]
**Other agents: Gulf War**	Pyridostigmine bromide PB, N,N-diethyl-m-toluamide DEET, permethrinPER and chlorpyrifos	GWI causing chronic pain, fatigue, sleep disturbances, cognitive problems	Ketamine for treating GWI-associated neuropsychiatric disorders	[5,42,44]

**Table 3:** MIP-based sensors for organophosphate agents, including CWAs, OPPs, and simulants and their characteristics.

Sensor Type/Detection Method	Polymerization Method	Electrode Modification	Target Molecule	LOD	Linear Range	Real Samples	Recovery Rate (%)	Refs.
Fluorescence combined with phosphatase-like nanozyme	One-pot reverse microemulsion	Gold nanoclusters (AuNCs) with MIPs polydopamine (PDA) and hollow CeO_2_ nanospheres CeO_2_@PDA@AuNCs-MIPs	Methyl-paraoxon	0.15 nM	0.45–125 nM	River and tap water	93.06–102.22	[51]
Colorimetric	Photo-polymerization	MIP-AchE inhibition	Cyantraniliprole Insecticides	4.1 ppm	15–50 ppm	Spiked melon	86.00–105.55	[52]
Colorimetric/fluorometric enzymatic	Chemiluminescence	Molybdenum disulfide/zirconium-based MOF MoS_2_@MIP-202(Zr) nanocomposite (NC)	Diazinon	0.12 nmol L^−1^	0.5–300.0 nmol L^−1^	Real water samples, river	95–102.5	[53]
Chemiluminescence (CL)	Bulk polymerization	MIP-based microtitration CL	Coumaphos, fenthion, chlorpyrifos, parathion, diazinon, fenchlorphos, and fenitrothion	1–3 pg mL^−1^	1–20 ng mL^−1^	Milk samples	Intraday86.1–86.5 and interday83.6–94.2	[54]
SAW gas/adsorption	Sol-gel polymerization	Piezoelectric/mesoporous SiO_2_MIP	Dimethyl methylphosphonateDMMP	80 ppb	80 ppb	NA *	NA *	[55]
Electrochemical/CV, DPV, EIS	Electropolymerization	MIP(O-PPy)/GCE	Profenofos	1 nM	1.0 × 10^−9^–5.0 × 10^−6^ M	Sweet pepper samples	108	[56]
Electrochemical/ CV, DPV	Surface and Electropolymerization	polyaniline nanomaterials (PANIs)-MIPs/GC	Parathion	0.011 μM	0.034–18.67 μM	Vegetables pakchoi, radish, lettuce, brassica chinensis, spinach, cabbage	98.2–100.1	[57]
Electrochemical/CV, EIS	Electropolymerization	Manganese oxide nanowires/two-dimensional molybdenum titanium carbide MXene (MnO_2_NWs@Mo_2_TiC_2_MXene)/GCE	Fenitrothion	3.0 × 10^−10^ mol L^−1^	1.0 × 10^−9^–2.0 × 10^−8^ mol L^−1^	White flour samples	Close to 100	[58]
Electrochemical/CV, EIS	Surface and Electropolymerization	Polythiophene copolymer loaded on—MWCNTs	Chlorpyrifos	4.0 pM	0.02–1000 nM	Vegetable samples	NA *	[59]
Non-Enzymatic Biomimetic Electrochemical/CV, DPV, EIS	Electropolymerization	2-aminothiophenol complex mixed with AuNPs/Au-SPE	Fenthion	0.05 mgkg^−1^	0.01–17.3 µg-mL^−1^	Olive oil samples	NA *	[60]
Photo electrochemical/CV	Electropolymerization	Carbon QD-modified titanium dioxide (MIP/C/TiO_2_NTs)	Triazophos	0.03 nM	0.1–1000 nM	Dongjiang River, drinking water and tap water	102–107	[61]
Electrochemical /DPV	Electropolymerization	-ZnO-hollow spheres (ZnOHS)—MIP/GCE	Methyl-parathion	0.5 × 10^−9^ mol L^−1^	5 × 10^−9^–0.1 × 10^−4^ mol L^−1^	Green beans, strawberry, tomato, and cabbage	90.4–91.9 and 96.3	[62]
Electrochemical /DPV	Electropolymerization	MIP/Cu-MOF/rGO/AuNPs/GCE	Phosmet	7.2 × 10^−15^ M	1.00 × 10^−14^–5.00 × 10^−7^ M	Apples, Cucumbers	94.2–106.5	[63]
Electrochemical /CV, EIS	Electropolymerization	MIP/Co_3_O_4_ nanowire and core-shell Co_3_O_4_@MOF-74 nanocomposite	Fenamiphos	3.0 × 10^−12^ M	10^−11^–10^−9^ M	Orange juice	99.65–100.48	[64]
Electrochemiluminescence (ECL)	Electropolymerization	MIP gold copper doped Tb-MOFs (Au@Cu:Tb-MOFs)/GCE	Chlorpyrifos	0.083 pM	0.285–0.285 × 10^6^ pM	Apples and cabbage samples	97.83–103.62	[65]
Electrochemiluminescence (ECL)	Electropolymerization	Flake-like nanocomposites Au@Cu:ZIF-8	Malathion	0.18 pgmL^−1^	10 pgmL^−1^ to 0.1 μgmL^−1^	Food and agricultural products	91.94–104.50	[66]
Electrochemical/ CV, EIS	Thermal polymerization	MIP based on4-ABA and TEOS/GCE	Ethyl methylphosphonic acid	2.75 × 10^−11^ M (standard), 2.11 × 10^−11^ M (urine), and 2.36 × 10^−11^ M (plasma)	1.0 × 10^–10^– 2.5 × 10^–9^ (standard), 1.0 × 10^–10^–2.5 × 10^–9^ (urine); 1.0 × 10^–10^–1.0 × 10^–9^ (plasma)	Human plasma and urine	99.86–101.30 in urine; 100.62–101.08 in plasma	[67]
Electrochemical/CV, EIS	Emulsion polymerization	MIP based on 4-ABA and TEOS/GCE-	Parathion	1.86 × 10^−8^ mol L^−1^	10^−8^–10^−4^ mol L^−1^	Tap and lake water	97–99 in tap water; 94–96 in lake water	[68]
Biomarkers POCT—Test strip/(MIPs)-based LFA strategy	Bulk	Sample pad, conjugate, absorption and backing pad/AuNPs, MIPs, and MTs	Thiodiglycol	0.41 pgmL^−1^	10.0 pgmL^−1^–10,000.0 ng mL^−1^	Urine samples	96.2–105.4	[69]
Biomarkers POCT—test strip/(MIPs)-based LFA strategy	Electrostatic assemblies of MIPs on the surface of PEI/PVA NFs membranes.	MIPs- PEI-PVA electrospun nanofiber membranes and AuNPs	Thiodiglycol	38 pgmL^−1^	0.1 ng mL^−1^–1.0 µg mL^−1^	Urine samples	105–111.6	[70]
Biomarkers POCT—test strip/(MIPs)-based LFA strategy	TDG was combined with the MAA through hydrogen bonding.	Coating MIPs supported on an NC membrane to obtain MIM and AuNPs	Thiodiglycol	1.0 ng mL^−1^–100.0 μg mL^−1^	0.23 ng mL^−1^	Urine samples	97.9–102	[71]

* NA not available.

**Table 4:** MIPs for explosives detection.

Sensor Type/ Detection Method	Preparation Method	Substrate/ Sensitive Material	Target	LoD	Linearity Range	Refs.
**Photoluminescence**	Deposited by spin-coating	CsPbBr3 nanocrystals (NCs) embedded in a polycaprolactone (PCL) matrix	Vapors of 3-nitrotoluene (3-NT) and nitromethane (NM)	0.218 mg mL^−1^	10^−9^–10^−3^ M 3-NT-	[103]
**Fluorescence**	Sol–gel process	Core-shell structure (MOF, Mg, N-CDs,r-CdTe), 3-amino-propyrtriethoxysilane (functional monomer) and tetraethyl orthosilicate (cross-linker)	Picric acid(2,4,6-trinitrophenol)	0.56 μM	1–100 μM	[104]
**Electrochemical/CV**	Electro polymerization	polycarbazole (PCz) films decorated with gold nanoparticles	Triacetone triperoxide (TATP) and hexamethylene triperoxide diamine (HMTD)	15 μgL^−1^	0.1−1.0 mg L^−1^	[105]
**Electrochemical/DPV**	Electrochemical polymerization	polycarbazole (PCz) deposited on Pt and -GCE-	Picric acid(2, 4, 6-trinitrophenol)	0.26 mM (MIP PCz/Pt) 0.57 mM (MIP PCz/GCE)	0.1–0.9 mM picric acid	[106]
**Electrochemical**	Electrochemical polymerization	trimesic acid and 3,4-ethylene-dioxythiophene were copolymerized on a tailormade laser-induced graphene electrode	2,4,6-Trinitrotolueen (TNT), 2,4,6-trinitrophenol (TNP), 2,4-dinitrotoluene (DNT), 1,3,5-trinitrobenzene (TNB), 2,4-dinitrophenol (DNP), and 1,3-dinitrobenzene (DNB)	TNT—1.95 ppbTNP—3.06 ppbDNT—2.49 ppbTNB—1.67 ppbDNP -1.94 ppbDNB—4.56 ppb	10 ppb–1000 ppb and1000 ppb–5000 ppb	[107]

**Table 7:** Current and emerging approaches for detecting biological agents.

Biological Agents	Detection Methods	References
**Pathogens**	Culture and colony counting, Immunology-based methods (PCR, lateral flow, ELISA, biosensors, fluorescence immunoassay, chemiluminescence assay, electrochemical immunoassay, SPR, fiber optic sensor, microfluidic biochip), MIPs, MOFs, etc.	[18,180,181,182,183,184,185,186,187]
**Viruses**	Cell culturing, PCR, ELISA, Flow cytometry, Biosensors Fluorescence, Raman and mass spectroscopy, NMR, SPR, Electrochemistry, HPLC, GC–MS, Electrogenerated ECL, ELISA, MIPs	[188,189,190,191,192,193,194,195]
**Toxins**	Radioimmunoassay, ELISA, Lateral flow, ECL, Biosensors, Fluorescence, Förster resonance energy transfer (FRET)	[194,196,197,198,199,200]
**Parasites**	Fluorescent microscopy, Flow cytometry, Automated blood cell analyzers, Serology antibody detection, Molecular methods, Laser desorption mass spectrometry, ELISA, Indirect fluorescence antibody test (IFAT)	[201,202,203,204,205,206]

**Table 8:** Prevalent biological warfare agents (BWAs).

Pathogen	Main Properties/Toxicology	Treatment /Therapy	Refs.
***Bacillus anthracis BWA—cat. A***	Gram-positive, rod-shaped bacteria that is an obligate, endospore-forming pathogen; spore form or vegetative form; skin (lesions) represents the main route to enter the organism; responsible for cutaneous, pulmonary, and gastrointestinal anthrax; symptoms: necrotic lesions, fever, nausea, vomiting, respiratory distress.	Anthrax vaccines and β-lactam antibiotics (e.g., penicillin), ciprofloxacin, doxycycline	[213]
***Clostridium*** ***Botulinum*** ***BWA—cat. A***	Anaerobic spore-forming gram-positive bacillus, rod-shaped neurotoxin; spores are highly resistant to heat, light, and drying; it blocks acetylcholine release across nerve synapses, leading to muscular paralysis and potentially death.	Antitoxins and antibiotics	[214]
***Yersinia pestis*** ***BWA—cat. A***	A nonmotile, gram-negative, facultative anaerobic, non-spore-forming, rod-shaped coccobacillus bacteria; transmission: flea bites, respiratory droplets; it is the causative agent of bubonic plague; bubonic, septicemic, or pneumonic forms.	Antibiotics (streptomycin, gentamicin)	[215]
***Variola virus*** ***BWA—cat. A***	Brick-shaped enveloped virus; the causative agent of smallpox; most frequently transmitted by droplet infection; symptoms: fever, rash, vomiting, skin blisters leaving scars.	Smallpox vaccines (eradicated globally)	[216]
***Francisella tularensis*** ***BWA—cat. A***	Intracellular, cytosolic, gram-negative, bacterial pathogen, often leads to a fatal disease called tularemia; symptoms include fever, headache, muscle aches, swollen lymph nodes, mouth ulcers, etc.	Antibiotics (streptomycin, gentamicin, doxycycline, ciprofloxacin)	[217]
***Ebola*** ***Virus*** ***BWA—cat. A***	Single-stranded RNA virus belonging to the *Filoviridae* family along with *Marburg virus*; *filoviruses* are enveloped, non-segmented RNA viruses with filamentous particles; *Ebola virus* causes a highly lethal hemorrhagic fever (the virus invades and kills the endothelial cells that line small blood vessels); symptoms: fever, fatigue, abdominal pain, severe headache, vomiting, diarrhea, myalgia, arthralgia, weakness, and hemorrhages.	Antiviral, monoclonal antibody treatments	[218]
***Marburg virus*** ***BWA—cat. A***	Hemorrhagic fever virus belonging to the family *Filoviridae* along with Ebola and has a single-stranded, negative-sense RNA genome; these viruses produce hemorrhagic shock syndrome and visceral organ necrosis.	Monoclonal antibodies	[219]
***Arenaviridae (Lassa, Machupo)*** ***BWA—cat. B***	Enveloped RNA viruses with bi-segmented genome with ambisense coding strategy; arenavirus infections could result in severe illness and death: Lassa fever (Sub-Saharan Africa), Machupo (South America).	Supportive care, ribavirin (Lassa)	[220]
***Brucella*** ***BWA—cat. B***	Gram-negative, coccobacilli-shaped bacterium; symptoms of brucellosis: severe fever (Malta fever), sweating, fatigue, headache, joint pain, endocarditis, and neurological complications.	Rifampin, doxycycline, streptomycin, no human vaccines available	[213]
***Vibrio cholerae*** ***BWA—cat. B***	Comma-shaped gram-negative bacterium; *Vibrio cholerae* is the aetiologic agent of cholera, a profound secretory diarrheal illness associated with the rapid onset of dehydration and hypovolemia.	Electrolytes, furazolidone, ampicillin, erythromycin, and fluoroquinolones	[221]
***Salmonellae*** ***BWA—cat.B***	Gram-negative rod-shaped bacteria; salmonella infections cause gastroenteritis, abdominal pain, fever, vomiting, nausea, diarrhea	Electrolytes, loperamide, cephalosporins	[222]
***Coxiella burnetii*** ***BWA—cat. B***	Pleomorphic, gram-negative intracellular bacterium generating Q fever (coxiellosis); symptoms: high fever, myalgia, malaise, nonproductive cough	Doxycycline, hydroxychloroquine	[223]
***Ricin toxin*** ***BWA—cat. B***	A type 2 ribosome-inactivating protein (heterodimer glycoprotein) isolated from the beans of the castor oil plant (*Ricinus communis*) leads to fluid and protein leakage and tissue edema, causing so-called ‘vascular leak syndrome’; inhalational exposure is the primary concern in terms of its use as a potential bioterrorism agent.	Vaccination (prophylaxis) and antitoxin (therapeutic) approaches	[224]
***Rickettsia prowazekii*** ***BWA—cat. B***	A small, gram-negative, obligately intracellular, rod-shaped bacterium leading to epidemic typhus fever; it remains highly infectious after drying in media with high osmolarity and has been weaponized so that it could be used as an agent of bioterrorism; symptoms include fever, headache, prostration, small and pink macules, and hemorrhagic rash.	Doxycycline, chloramphenicol	[225]

## Data Availability

Not applicable.

## Abbreviations

A-230	Methyl-(bis(diethylamino)methylene)phosphonamidofluoridate
A-232	Methyl (1-(diethylamino)ethylidene)phosphoramidofluoridate
A-234	Ethyl (1-(diethylamino)ethylidene)phosphoramidofluoridate
A-242	O-n-Decyl N-(1-(di-n-decylamino)-n-decylidene)phosphoramidofluoridate
A-262	Guanidine-bearing phosphoramidofluoridate structure
ABA	4-Aminobenzoic acid
AchE	Acetylcholinesterase
AH-7921	[3,4-dichloro-N-[[1(dimethylamino) cyclohexyl]methyl] benzamide]
ANFO	Ammonium nitrate/fuel oil
ATR-FTIR	Total reflectance Fourier transform-infrared
NIR	Near-infrared spectroscopy
Au-E	Gold disk electrode
AuNCs	Gold nanoclusters
AuNPs	Gold nanoparticles
*B. anthracis*, *B. subtilis*	*Bacillus* species
*B. cereus*	*Bacillus cereus*
BPAEC	Bovine pulmonary artery endothelial cells
*B. suis*, *B. melitensis*, *B. abortus*	*Brucella* species
BUP	Buprenorphine
BWAs	Biological warfare agents
BWC	Biological Weapons Convention
BZ3	Quinuclidinyl benzilate or 1-azabicyclo[2.2.2]octan-3-yl hydroxy(diphenyl)acetate
*C. botulinum*	*Clostridium botulinum* toxin
*C. psittaci*	*Chlamydia psittaci*
CBD	Cannabidiol
CG	Phosgene or Carbonyl dichloride
Cl	Chlorine
CN	ω-Chloroacetophenone
CPE	Carbon paste electrode
CPS	Carboxylic-terminated polystyrene
CR	Dibenz[b,f][1,4]oxazepin
CS	α-chlorbenzylidenemalonitrile
CV	Cyclic voltammetry
CWAs	Chemical warfare agents
DC	Diphenylarsinous cyanide
DEET	N,N-diethyl-meta-toluamide
DFP	Diisopropylfluorophosphate
DMMP	Dimethyl Methylphosphonate
DNB	1,3-Dinitrobenzene
DNP	2,4-Dinitrophenol
DNT	2,4-Dinitrotoluene
DPV	Differential pulse voltammetry
ECL	Electro-chemiluminescence
EDOT	3, 4-Ethylenedioxythiophene
EGDMA	Ethylene glycol dimethacrylate
EIS	Electrochemical Impedance Spectroscopy
ELISA	Enzyme-linked immunosorbent assays
EMPA	Ethyl methyl phosphonic acid
ErGO	Reduced graphene oxide
*Escherichia coli* species	*E. coli*
EV71	Enterovirus 71
FRET	Förster resonance energy transfer
FXR	Farnesoid X receptor
GA	Tabun O-Ethyl N,N-dimethylphosphoramidocyanidate
GB	Sarin or O-Isopropyl methylphosphonofluoridate
GC	Gas chromatography
GCE	Glassy carbon electrode
GC-MS	Gas chromatography-mass spectrometry
GD	Soman or O-Pinacolyl methylphosphonofluoridate
GO	Graphene oxide
GQDs	Graphene quantum dots
GWI	Gulf War Illness
HCN	Hydrogen cyanide
HD	Sulfur mustard or 2-Chloroethylchloromethylsulfide
HHC	Hexahydrocannabinol
HMX	Octogen
HPLC	High-pressure liquid chromatography
HPLC	MS/MS High-performance liquid chromatography-tandem mass spectrometry
HRMS	High-resolution mass spectrometry
IC	MS/MS Ion chromatography-tandem mass spectrometry
IF	Imprinting factor
IFAT	Indirect fluorescence antibody test
IMS	Ion mobility spectroscopy
IPD-IC	Indirect photometric detection ion chromatography
ISE	Ion-selective electrode
ITO	Indium tin oxide
L	Lewisite 1 or 2-Chlorovinyldichloroarsine
LC	MS/MS Liquid chromatography-tandem mass spectrometry
LFA	Lateral flow assay
LSD	Lysergic acid diethylamide
LSV	Linear sweep voltammetry
MAA	Methacrylic acid
MABs	Monoclonal Antibodies
MBA	N,N′-Methylenebisacrylamide
MDMA	Ecstasy or 3,4-methylenedioxymethamphetamine
MDPEA	3,4-Methylenedioxyphenethylamine
MESNA	Sodium 2-mercaptoethane sulfonate
METH	Methamphetamine
MI	Molecular imprinting
MIM	Molecularly imprinted membrane
MIPs	Molecular imprinted polymers
MMIP	Magnetic molecularly imprinted polymers
MOF	Metal–organic frameworks
MT-45	[1-cyclohexyl-4-(1,2-diphenylethyl)piperazine]
MTs	Metallothioneins
MWCNTs	Multi-wall carbon nanotubes
nanoMIPs	Nano-sized molecularly imprinted polymers
NC	Membrane nitrocellulose membrane
NCHS	National Center for Health Statistics
NG	Nitroglycerine
NLX	Naloxone
NM	Nitrogen mustard or 2-Chloro-N,N-bis(2-chloroethyl)ethanamine
NTO	3-nitro-1,2,4-triazol-5-one
OC	Oleoresin of capsicum or pepper spray
OP	Organophosphate
OPCW	Organization for Prohibition of Chemical Weapons
PSI-MS	Paper spray ionization mass spectrometry
PB	Pyridostigmine bromide
PDA	Polydopamine
PEI	Polyethyleneimine
PER	Permethrin
PETN	pentaerythritol tetranitrate
PHO	Pholcodine (3-(2-morpholinoethyl)morphine)
POC	Point-of-care
*Ps. Aeruginosa*	*Pseudomonas aeruginosa*
PSIMS	Paper spray ionization mass spectrometry
PANI	Polyaniline
PPy	Polypyrole
PVA	Poly(vinylalcohol)
QCM	Quartz crystal microbalance
QDs	Quantum dots
RDX	Hexogen
RGO	Reduced graphene oxide
RS	Raman spectroscopy/scattering
SAW	Surface acoustic wave
SDC	Substrate displacement colorimetry
SF	Cyclosarin or cyclohexyl methylphosphonofluoridate
SIP	Surface imprinted polymer
SM	Mustard gas or Bis(2-chloroethyl)sulfide
SPCE	Screen-Printed Carbon Electrode
SPE	Solid phase extraction
SPEs	Screen-Printed Electrodes
SPME	Solid phase microextraction
SPPE	Screen-printed platinum electrodes
*S. Paratyphi*	*Salmonella Paratyphi*
*S. aureus*, *S. epidermidis*	*Staphylococcus* species
*S. pneumoniae*	*Streptococcus pneumoniae*
STX	Saxitoxin
SUF	Sufentanil
SWV	Square Wave Voltammetry
SWV	Square wave voltammetry
TDG	Thiodiglycol
TEOS	Tetraethyl orthosilicate
THC	Trans-Δ9-tetrahydrocannabinol
THCP	Tetrahydrocannabiphorol
TNB	1,3,5-Trinitrobenzene
TNP	2,4,6-Trinitrophenol
TNT	2,4,6-Trinitrotoluene
U-47700	[3,4-dichloro-N-[(1R,2R)-2-(dimethylamino)yclohexyl]-N-methylbenzamide]
U-48800	[trans-2-(2,4-dichlorophenyl)-N-2-(dimethylamino)cyclohexyl)-N-methylacetamide,monohydrochloride]
U-49900	[3,4-dichloro-N-(2-(diethylamino)cyclohexyl)-N-methylbenzamide]
U-50488	[trans-3,4-dichloro-N-methyl-N-[ 2-(1-pyrrolidinyl) cyclohexyl]-benzeneacetamide]
*V. cholera*	*Vibrio cholerae*
VX	O-Ethyl S-2-diisopropylaminoethyl methylphosphonothiolate
*Y. pestis*	*Yersinia pestis*
ZnO	Zinc oxide
